# Low glucose availability potentiates the effects of metformin on model T cell activation and exhaustion markers *in vitro*

**DOI:** 10.3389/fendo.2023.1216193

**Published:** 2023-12-05

**Authors:** Jernej Repas, Lea Peternel, Harald Sourij, Mojca Pavlin

**Affiliations:** ^1^ Institute of Biophysics, Faculty of Medicine, University of Ljubljana, Ljubljana, Slovenia; ^2^ Trials Unit for Interdisciplinary Metabolic Medicine, Division of Endocrinology and Diabetology, Medical University Graz, Graz, Austria; ^3^ Group for Nano- and Biotechnological Applications, Faculty of Electrical Engineering, University of Ljubljana, Ljubljana, Slovenia

**Keywords:** T cells, metformin, glucose level, 2-deoxy-D-glucose, T cell exhaustion, PD-1/PD-L1 axis

## Abstract

Modulation of immune cell metabolism is one of promising strategies to improve cancer immunotherapies. Metformin is an anti-diabetic drug with potential anti-cancer effects, ranging from normalization of blood glucose and insulin levels, direct anti-proliferative effects on cancer cells to emerging immunomodulatory effects on anti-tumor immunity. Metformin can reduce tumor hypoxia and PD-L1 expression, as well as normalize or improve T cell function and potentiate the effect of immune checkpoint inhibitors, making it a promising adjuvant to immunotherapy of tumors with poor response such as triple negative breast cancer (TNBC). However, although the effects of metformin on cancer cells are glucose-dependent, the role of glucose in modulating its effect on T cells has not been systematically studied. We thus investigated the effect of metformin as a function of glucose level on Jurkat cell and PBMC T cell models *in vitro*. While low metformin concentrations had little effect on T cell function, high concentration reduced proliferation and IFN-γ secretion in both models and induced a shift in T cell populations from memory to effector subsets. The PD-1/CD69 ratio was improved by high metformin in T cells from PBMC. Low glucose and metformin synergistically reduced PD-1 and CD69 expression and IFN-γ secretion in T cells from PBMC. Low glucose level itself suppressed Jurkat cell function due to their limited metabolic plasticity, but had limited effects on T cells from PBMC apart from reduced proliferation. Conversely, high glucose did not strongly affect either T cell model. Metformin in combination with glycolysis inhibitor 2-deoxy-D-glucose (2DG) reduced PD-1 in Jurkat cells, but also strongly suppressed their function. However, low, physiologically achievable 2DG concentration itself reduced PD-1 while mostly maintaining IL-2 secretion and, interestingly, even strongly increased IFN-γ secretion regardless of glucose level. Overall, glucose metabolism can importantly influence some of the effects of metformin on T cell functionality in the tumor microenvironment. Additionally, we show that 2DG could potentially improve the anti-tumor T cell response.

## 1 Introduction

In the recent years, the anti-diabetic drug metformin has received a lot of attention as a potential anti-cancer agent (1), with its use associated with decreased incidence of several cancers (1, 2) and improved outcomes in colorectal and lung cancer (3, 4). However, some anti-cancer effects of metformin are still controversial (5) and, in parallel, its mechanisms of action are still being investigated (1, 5, 6). Metformin was shown to have both systemic effects on glucose and insulin levels (7) and to directly suppress cancer cell respiration via NADH oxidase (7–9). Recently, immunomodulatory effects of metformin have been identified (10, 11), with direct effects on both the immune and cancer cells, as well as the tumor microenvironment (12). Metformin improves tumor hypoxia (13) and T cell survival in hypoxic environment (14), as well as decreases the number of infiltrating T_reg_ cells (15) and myeloid-derived suppressor cells (16). Additionally, metformin can induce PD-L1 degradation in triple negative breast cancer (17) and synergize with immune checkpoint inhibitors (12, 18–21). Recently, metformin has been shown to directly normalize or improve the antigen-specific effector functions of T cells (22–26) which are key factors of anti-tumor immunity. On the other hand, metformin can suppress T cell activation and function in the context of autoimmune diseases (27–33) and viral hepatitis (34). Altogether, the potential immunomodulatory antitumor effects of metformin are still under investigation, necessitating further exploration of the determinants of its effects on T cells.

While metformin acts to normalize elevated systemic glucose levels, its direct anti-proliferative effects on cancer cells are dependent on nutrient, particularly glucose levels (9, 35), since glycolysis represents a major source of ATP and biosynthetic intermediates for fast proliferating cancer cells (36). Similarly, activated T cells also have upregulated glycolysis via mTOR and PI3K/Akt signaling (37–41) in addition to increased oxidative phosphorylation and ROS generation (42). Metabolic changes are deeply linked to T cell differentiation and subtype specification (39, 43, 44), as well as their effector functions, with glycolysis involved in INF-γ secretion (42, 45) and glutamine metabolism in Th17 cell function (46). This directly implicates strong influence of nutrient availability on these processes, with glucose for example required for IFN-γ translation (45). Glucose levels could be particularly important in diabetic cancer patients, ranging from >11 mM (in the blood of unsuccessfully treated diabetic patients) to nearly 0 mM in the tumor core (47, 48). The excess glucose consumption by tumor cells is a form of tumor suppression of T cell responses (49, 50), which require T cell metabolic fitness with sufficient mitochondrial mass (51) and considerable metabolic plasticity (52) to function in such low-glucose environments. The inhibition of respiration by metformin could reduce this metabolic plasticity (25), and the combination of metformin and glycolysis inhibitor 2-deoxy-D-glucose (2DG) significantly suppressed T cell functions (53). However, metformin treatment can also lead to increased T cell mitochondrial mass (34, 54, 55), so it is necessary to investigate the effect of metformin on T cells at low as well as normal and elevated glucose levels.

Additionally, surface protein glycosylation directly linked to glycolysis was shown to importantly affect the expression and function of several T cell immune receptors and their ligands (56). Metformin can induce abnormal PD-L1 glycosylation and degradation (17), and targeting N-glycosylation by 2DG or the combination of metformin and 2DG can decrease PD-L1 expression on tumor cells (57–60). Interestingly, metformin can normalize PD-L1 expression increased by low glucose (60, 61). As PD-1 function is also affected by glycosylation (62–64), it could similarly be affected by metformin and glucose availability, but little research has been done on the topic.

Despite the clear rationale for glucose level modulating the effects of metformin on T cells, very few studies have so far explored this question. Glucose concentration was very recently found by Chao et al. to determine the reliance of T cells on mitochondrial metabolism to produce IFN-γ, but only low metformin concentrations and 3-4 mM versus 12 mM glucose were used (25). Moreover, this and another study using *in vivo* glucose supplementation with metformin treatment only used mouse CD8+ T cells (25, 26). Additionally, despite the emerging chronic effects of diabetes and hyperglycemia on T cell function (65), the shorter term impact of high glucose concentrations (11-25 mM) routinely used in T cell culture media as opposed to physiological (~5 mM) levels has hardly been studied in a controlled *in vitro* setting, especially with metformin treatment. Very little is therefore known about the effects of metformin on human T cells and CD4+ T cells in particular at glucose concentrations <1 mM and >12 mM, both likely to occur in cancer and/or diabetic patients.

In the present study, we therefore aimed to understand how the effects of metformin on the function of T cells are modulated by glucose concentration, using the Jurkat cell line and peripheral blood mononuclear cells (PBMC) as T cell models. We investigated the effects of metformin on survival, proliferation, activation, metabolism and effector functions of model T cells. Finally, we evaluated the effect of metformin and 2DG on PD-1 expression in order to understand potential implications for anti-tumor T cell immunity.

## 2 Materials and methods

### 2.1 Cell culture, PBMC isolation and treatments

Jurkat cells were acquired from ATCC and maintained in ATCC-modified RPMI 1640 medium with 25 mM glucose, 2 mM glutamine and 1 mM pyruvate (Gibco, Thermo Fisher, Waltham, MA, USA) supplemented with 10% FBS (Sigma-Aldrich, Saint Louis, MO, USA). All experiments were performed in RPMI 1640 medium (Genaxxon bioscience GmbH, Ulm, Germany) supplemented with 10% FBS and 2 mM glutamine (Sigma-Aldrich) unless otherwise indicated. The seeding densities were 5×10^5/mL, 2.5×10^5/mL and 1.25×10^5/mL for 24 h, 48 h and 72h treatment, respectively. The medium for experiments was also supplemented with 25 mM, 5.6 mM, 0.56 mM or 0 mM glucose, and the cells treated with 0.03 mM – 5 mM metformin for 24h to 72h as indicated. Where indicated, the medium was additionally supplemented with 50 pM to 1 μM human insulin (I9278, Sigma-Aldrich, Saint Louis, MO, USA).

Peripheral blood mononuclear cells (PBMC) were isolated from buffy coats of four healthy donors by ficoll-gradient centrifugation. Briefly, peripheral blood was allowed to cool to room temperature for 30 min, diluted with 2 mM EDTA in phosphate buffered saline (PBS) and carefully placed on a layer of Ficoll-Paque^®^ Premium (GE Healthcare Bio-Sciences AB, Uppsala, Sweden). Separation was performed by centrifugation at 400 g for 40 min without active braking. The resulting buffy coats were aspirated and washed three times with 2 mM EDTA in 1x PBS. PBMC cells were suspended in complete RPMI medium (RPMI 1640 medium (Genaxxon bioscience GmbH, Ulm, Germany) supplemented with 10% autologous serum and 2 mM glutamine), counted and diluted to 2×10^6^ cells/mL. 300 µL of this suspension was seeded on 48-well cell culture plates and 300 µL of complete RPMI medium with either 50 mM, 11.2 mM or 1.12 mM glucose and either 0 mM or 10 mM metformin was added per well to achieve final glucose concentrations of 25 mM, 5.56 mM or 0.56 mM, and metformin concentration of 0 mM or 5 mM as indicated. The cells were treated with AMPK activator A 769662 (Abcam, Cambridge, UK) and glycolysis inhibitor 2-deoxy-D-glucose (2DG) (Sigma-Aldrich, Saint Louis, MO, USA) in the same manner with the final concentration of 70 µM and 4.8 mM, respectively. The cells were treated for 72 h, after which the cells were harvested by centrifugation, the medium collected for cytokine secretion and the cells used for further analysis.

In some experiments, the T cells were activated for the duration of the treatment. The cell culture plates were coated with 150 μL/well of 10 μg/mL (T cells from PBMC) or 20 μg/mL (Jurkat) αCD3 antibodies (300438, Biolegend, San Diego, CA, USA) in 1x PBS for at least 2h at 37°C and washed three times with 1×PBS prior to PBMC seeding. αCD28 antibodies (302934, Biolegend) were added to the final concentration of 5 μg/mL (T cells from PBMC) or 10 μg/mL(Jurkat) for 24h for Jurkat cells and IL-2 secretion or 72h for other experiments on T cells from PBMC. For some Jurkat cell experiments, 25 ng/mL PMA and 1.0 μM ionomycin (both from Sigma-Aldrich, Saint Louis, MO, USA) were added for 24h instead.

### 2.2 Total cell number, proliferation and the percentage of dead cells

The total number of Jurkat cells was determined spectrofluorimetrically by staining the DNA with Hoechst 33342 by a previously described protocol (60) adapted for suspension cells. Briefly, after treatment, 25 µg/mL Hoechst 33342 was added to the cells for 30 min at 37°C. The washing step was omitted and the fluorescence intensity measured at 350 nm excitation and 461 nm emission using a Tecan Infinite 200 microplate reader (Tecan, Männerdorf, Switzerland). The background Hoechst 33342 fluorescence was subtracted and the results were normalized to untreated control cells (5.6 mM glucose, 2 mM glutamine, 0 mM metformin). The percentage of dead Jurkat cells was determined by propidium iodide (PI) staining. After treatment, the cells were harvested by centrifugation and resuspended in cold 1×PBS. 0.15 mM PI was added immediately before the measurement and the cells were analyzed on Attune NxT flow cytometer (Thermo Fisher, USA).

The proliferation of T cells from PBMC was determined by CFSE staining. An aliquot of isolated PBMC cells was washed separately for the final washing step, re-suspended in 1×PBS and stained with 0.25 μM CFSE (Biolegend, San Diego, CA, USA) for 20 min at 37°C. Staining was quenched by the addition of autologous serum containing medium, the cells washed by centrifugation, counted, seeded on 48-well plates coated with anti-CD3 antibodies, activated and treated as described above. After 72h treatment, cells were harvested by centrifugation, suspended in cold 1×PBS and analyzed on Attune NxT flow cytometer (Thermo Fisher, USA). The percentage of proliferated cells was defined as the percentage of cells with CFSE fluorescence between the peak at the highest CFSE fluorescence and the lowest peak of background fluorescence. The percentage of dead PBMC cells was determined by staining the cells with Zombie Yellow (Biolegend, San Diego, CA, USA) for 20 min at room temperature according to manufacturer’s instructions, followed by analysis on Attune NxT flow cytometer (Thermo Fisher Scientific, Waltham, MA, USA).

### 2.3 Real time metabolic assay

Jurkat cells were seeded on 24-well cell culture plates at 2.5 ×10^5^ cells/mL and treated for 48 h with 0.3 mM or 5 mM metformin in medium supplemented with 5.6 mM or 0 mM glucose as indicated. After treatment, cells were spun down and resuspended in Seahorse XF RPMI 1640-based Seahorse XF Glycolytic Rate Assay medium (2 mM glutamine, 1 mM HEPES, 0 mM pyruvate, 5.6 mM or 0 mM glucose) equilibrated to pH 7.4 and plated on Seahorse cell culture microplates covered with CellTak^®^ (Corning, Corning, NY, USA) at 100,000 cells in 0.1 mL per well. Plates were spun down at 200 g for 1 min and incubated at 37°C without CO_2_ for 15 min, after which 0.4 mL of the medium was added. After additional 30 min of incubation at 37°C without CO_2_, the Seahorse Mito Stress Assay was performed according to the manufacturer’s instructions using 1.5 μM oligomycin, 1.5 μM FCCP and 0.5 μM rotenone/antimycin A on the Seahorse XFe24 analyzer (Agilent Technologies, Santa Clara, CA, USA).

Glycolytic ATP production was calculated as glycolytic proton efflux rate according to equation: glycoATP Production Rate (pmol ATP/min) = glycoPER (pmol H+/min) = basalPER (pmol H+/min) – MitoPER (pmol H+/min) = basalPER – (basal OCR – OCR after rotenone/antimycin A) * 0.6. The oxidative phosphorylation ATP production rate (OxPhosATP) was calculated according to formula: OxPhosATP (pmol ATP/min) = (basal OCR – OCR after oligomycin) (pmol O_2_/min) * 2 (pmol O/pmol O_2_) * P/O (pmol ATP/pmol O_2_) assuming a P/O ratio of 2.75.

### 2.4 Cell surface markers and transcription factors expression

Jurkat cells were seeded on 12-well plates at 5 ×10^5^ cells/mL in complete RPMI with 5.6 mM or 0 mM glucose and treated with 0.3 mM or 5 mM metformin in media with 25 ng/mL PMA and 1.0 μM ionomycin and either 25 mM, 5.6 mM or 0 mM glucose for 24h as indicated. The cells were then harvested by centrifugation and stained with APC-conjugated anti-PD-1 (329908, Biolegend, San Diego, CA, USA) and Pacific Blue conjugated anti-CD69 (310920, Biolegend, San Diego, CA, USA) at room temperature for 20 min. Cells were then washed with PBS with 1% BSA, resuspended in PBS and analyzed on Attune NxT flow cytometer (Thermo Fisher Scientific, Waltham, MA, USA).

PBMC cells were isolated, cultured and treated as described above for 72h, after which they were harvested and stained at room temperature for 20 min with the following antibodies: APC/Cy7 conjugated anti-CD3 (300318), PerCP-Cy5.5 conjugated anti-CD4 (300530), FITC conjugated anti-CD8 (300906), PE-Cy7 conjugated anti-CD45RA (304126), Alexa^®^ 700-conjugated anti-CD197 (CCR7) (353244), APC-conjugated anti-PD-1 (329908), and Pacific Blue conjugated anti-CD69 (310920). All antibodies were obtained from Biolegend, San Diego, CA, USA. Cells were then washed with PBS with 1% BSA, resuspended in PBS and analyzed on Attune NxT flow cytometer. For staining of intracellular transcription factors, the cells were washed after performing the surface staining protocol, then fixed and permebilized using the True-Nuclear™ Transcription Factor Buffer Set (Biolegend, San Diego, CA, USA) according to manufacturer's instructions. The following antibodies were used for intracellular staining according to manufacturer's instructions: PE-conjugated anti-HIF-1α (359703, Biolegend), PE-conjugated anti-Eomes (566749, BD Biosciences, Franklin Lakes, NJ, USA), APC-conjugated anti-STAT3 (371805, Biolegend), BV421™-conjugated anti-T-bet (644832, Biolegend) and BV421™-conjugated anti-P-S6RP (608609, Biolegend). After staining, the cells were washed, resuspended in PBS and analyzed on Attune NxT flow cytometer. The gating strategy for all flow cytometry analyses are available in section 1.2 of the **Supplementary Material**.

### 2.5 Mitochondrial mass

Jurkat cells were seeded on 12-well plates at 1.25 ×10^5^ cells/mL in complete RPMI with different glucose concentrations and treated with 0.3 mM or 5 mM metformin for 72h as indicated. The cells were harvested by centrifugation and stained with either 7.5 μM nonyl-acridine orange (NAO) for 20 min or 50 nM Mitotracker Orange for 45 min at 37°C. Cells were then washed by centrifugation, resuspended in PBS and analyzed on Attune NxT flow cytometer (Thermo Fisher Scientific, Waltham, MA, USA).

### 2.6 Cytokine secretion

Jurkat cells were seeded on 12-well plates at 5 ×10^5^ cells/mL in complete RPMI with 5.6 mM or 0 mM glucose and treated with 0.3 mM or 5 mM metformin in media with 25 ng/mL PMA and 1.0 μM ionomycin and either 25 mM, 5.6 mM or 0 mM glucose for 24h as indicated. The cells were harvested by centrifugation and the supernatants collected and stored at -80°C until further analysis. The concentration of IL-2 and IFN-γ was determined using human IL-2 ELISA kit (88-7025, Invitrogen, Waltham, MA, USA) and human IFN-γ ELISA kit (88-7316, Invitrogen, Waltham, MA, USA) according to the manufacturer’s instructions.

### 2.7 Statistical analysis

The statistical analysis was performed using GraphPad Prism (v9; GraphPad Software, Inc., La Jolla, CA, USA). For Jurkat cells, the results were displayed as mean ± SEM of three biological replicates unless indicated otherwise. For PBMC cells, the results were displayed as median ± interquartile range unless indicated otherwise. The statistical significance of the effect of the two factors investigated (metformin and glucose unless stated otherwise) and their potential synergism was tested using two-way ANOVA with Dunnett or Šidak *post-hoc* test. When only one variable (eg. metformin) was investigated, one-way ANOVA with Dunnett *post-hoc* test was used. For experiments with PBMC, repeated measures two-way ANOVA or paired Student's t-test was used to account for considerable inter-donor variability. Unless indicated otherwise, p-value under 0.05 was considered statistically significant.

### 2.8 Ethics approval statement

The study was carried out in concordance with the Declaration of Helsinki and was approved by the National Medical Ethic Committee (approval number 0120-237/2018/14). The participants provided their written informed consent to participate in this study.

## 3 Results

### 3.1 Low glucose availability and metformin independently reduce cell number and synergistically increase cell death in Jurkat cell

**Figure 1:**
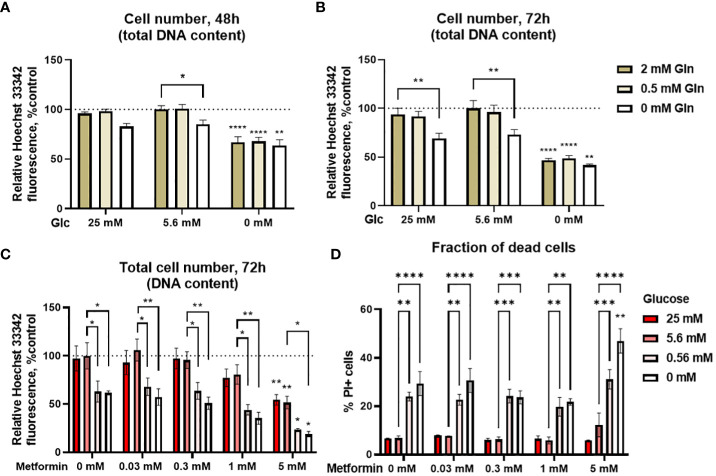
The effect of the metformin treatment and nutrient availability on Jurkat cell survival and proliferation. **(A, B)** Jurkat cells were grown in media with different glucose and glutamine (denoted by shades of green) concentrations. The total number of cells was determined after 48 h **(A)** or 72 h **(B)** by Hoechst 33342 staining. **(C, D)** Jurkat cells were grown in media with different glucose concentrations (denoted by shades of red) and treated with different metformin concentrations as indicated for 72 h. The total cell number **(C)** was determined by Hoechst 33342 staining and the percentage of dead cells **(D)** was determined by PI staining using flow cytometry. The mean ± SEM is shown for three independent experiments. *p<0.05, **p<0.01, ***p<0.001, ****p<0.0001 vs. 5.6 mM glucose at the same glutamine or metformin concentration unless indicated otherwise, as determined by two-way ANOVA with Dunnett’s *post-hoc* test. Two-way ANOVA did not confirm any synergism between the effects of glucose and glutamine **(A, B)**, or glucose and metformin **(C)** on the total cell number, but there was significant interaction between glucose and metformin on the percentage of PI+ cells **(D)**.

Glucose and glutamine are two major cellular fuels and carbon sources, so we first evaluated their effect on proliferation of Jurkat cells. We determined the total cell number spectrofluorimetrically with Hoechst 33342 staining as described previously (60). 5.6 mM glucose was used to mimic normal physiological conditions in the blood, 25 mM was used to model concentrations used in routine cell culture, while 0 mM (medium containing only glucose from serum) was used to mimic the conditions in the tumor microenvironment. For glutamine, 2 mM represented the standard cell culture conditions (control), 0.5 mM was used to mimic the physiological levels and 0 mM was used to represent the low nutrient conditions in the tumor microenvironment. Jurkat cells were mostly dependent on glucose, as their total cell number was significantly decreased in 0 mM glucose medium to about 65% of the control – medium with 5.6 mM glucose after 48h (**Figure 1A**) and about 50% of the control after 72h (**Figure 1B**) regardless of glutamine concentration. On the other hand, high glucose levels (25 mM) did not affect the cell number compared to the control. Conversely, glutamine availability had a limited effect, as the cell number was only reduced in 0 mM glutamine by 15% after 48h and 25% after 72h in the presence of glucose, while it had no effect at 0 mM glucose. There was no difference between 2 mM and 0.5 mM glutamine regardless of the glucose level. Overall, two-way ANOVA did not confirm a significant interaction between glucose and glutamine levels. Jurkat cells were thus mostly dependent on the glucose level, so we focused on the glucose availability in our subsequent experiments, while glutamine levels were kept at their standard cell culture (2 mM) level.

We next investigated how various glycemic levels modulate the effect of metformin on the proliferation of Jurkat cells by measuring the total cell number and the percentage of dead cells. Low metformin concentrations achievable in the plasma of patients (0.03 mM and 0.3 mM) had no apparent effect regardless of the glucose concentration after 72h treatment (**Figure 1C**). On the other hand, concentrations used to mimic cellular metformin accumulation with long term use decreased the total cell number in a dose-dependent manner to about 80% of control for 1 mM metformin (not significant) and 50% of control for 5 mM metformin when sufficient glucose was present. The same was true in the low glucose media (0.56 mM and 0 mM glucose) where 5 mM metformin reduced the total cell number from about 60-65% to about 25% and 20%, respectively. While the total cell number was further decreased by metformin treatment in low glucose, two-way ANOVA did not confirm any synergism between the effects of glucose and metformin. The same trends for both glucose and metformin were also observed after 48h treatment (**Supplementary Figure 1**).

Decreased total cell number can indicate either suppressed proliferation or cell death. We therefore determined the percentage of dead cells with PI staining. There was no increase in the percentage of dead cells with metformin when sufficient glucose was available (**Figure 1D**). Conversely, cell death was increased in medium with 0 mM or 0.56 mM glucose. There was no further increase in cell death with metformin concentrations up to 1 mM in these media. However, 5 mM metformin did significantly increase the percentage of dead cells at 0 mM glucose, and two-way ANOVA confirmed the synergistic effect of metformin and low glucose on the percentage of dead cells. Overall, the results indicate that the observed decrease in the total cell number with metformin treatment is mostly the result of decreased proliferation when sufficient glucose is available. On the other hand, low glucose availability can itself reduce Jurkat cell number, while also acting synergistically with high metformin concentrations to further increase Jurkat cell death.

### 3.2 Metformin reduces oxidative ATP production in Jurkat cells which cannot be compensated by increased glycolysis

**Figure 2:**
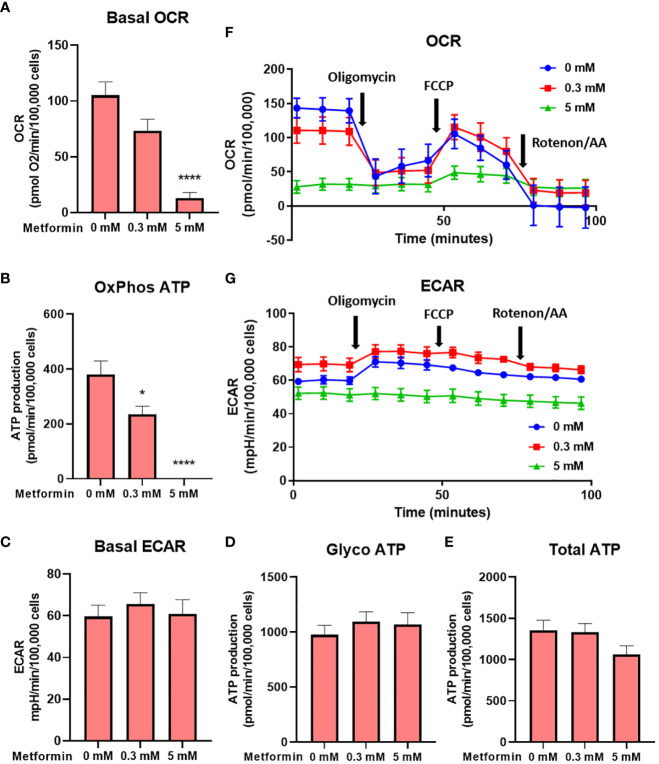
The effect of the metformin treatment on Jurkat cell energy metabolism. Jurkat cells were grown in media with 5.6 mM glucose and treated for 48h with 0 mM, 0.3 mM or 5 mM metformin as indicated. Baseline OCR **(A)** and ECAR **(C)** were determined using Seahorse Mito Stress Test assay. The ATP production from oxidative phosphorylation **(B)** and glycolysis **(D)**, as well as total ATP production **(E)** were calculated according to the manufacturer’s instructions for Seahorse Real Time ATP Assay. Mean ± SEM is shown for five independent experiments. *p < 0.05, ****p<0.0001, as determined by ANOVA with Dunnett’s *post-hoc* test. A representative time-course for OCR and ECAR are shown in **(F, G)**, respectively.

To confirm that the effect of metformin on reduced Jurkat cell proliferation is primarily caused by disrupted cellular bioenergetics (as opposed to other effects, e.g. redox imbalance), we next performed the Seahorse Mito Stress assay. We found that metformin reduced the oxygen consumption rate (OCR) in a dose-dependent manner, reaching about 75% of control level at 0.3 mM (not significant) and 15% of control levels at 5 mM (p<0.0001, **Figure 2A**). When correcting for sources of oxygen consumption not linked to ATP production, this translated to about 40% drop in ATP production through oxidative phosphorylation at 0.3 mM and its complete inhibition at 5 mM (**Figure 2B**). There was no significant compensatory increase in the extracellular acidification rate (ECAR) or glycolytic ATP production at either metformin concentration (**Figures 2C, D**). As a result, while the total ATP production was unchanged by 0.3 mM metformin, there was a trend (not significant) towards lower total ATP production with 5 mM metformin treatment (**Figure 2E**). The results therefore point to the limited ATP generation capacity of metformin-treated Jurkat cells that is contributing to reduced proliferation. A similar but much more pronounced (about 70%) drop in total ATP production was also observed in the medium without glucose where glycolysis was completely suppressed (**Supplementary Figure 2**), demonstrating the reliance of Jurkat cells on glycolysis and their lack of substantial spare capacity in either glycolysis or respiration.

### 3.3 Metformin and low glucose availability reduce Jurkat cell activation and effector function

**Figure 3:**
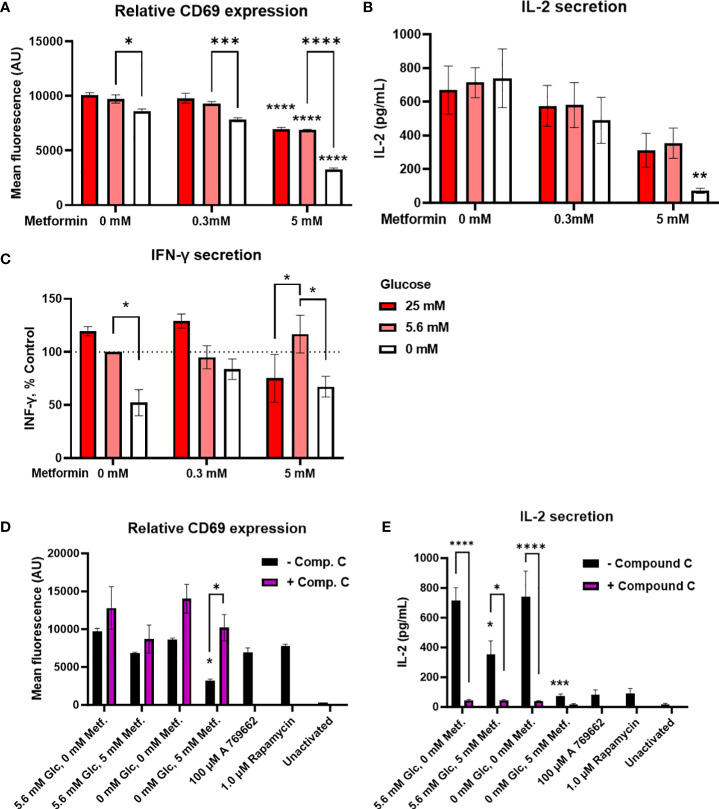
The effect of metformin as a function of glucose availability on Jurkat cell activation and effector functions. Jurkat cells were activated with anti-CD3/anti-CD28 antibodies **(A, B, D, E)** or PMA/ionomycin **(C)** and grown for 24 h in media with different glucose (denoted by shades of red) and metformin concentrations. **(D, E)** Jurkat cells were pretreated with 5μM compound C (AMPK inhibitor) for 30 min, followed by metformin treatment at 5.6 mM or 0 mM glucose for 24h. **(A, D)** Relative surface expression of CD69 was determined by flow cytometry. The concentration of IL-2 **(B, E)** and IFN-γ **(C)** in the medium was determined by ELISA. The mean ± SEM is shown for three **(A, C–E)** or four **(B)** independent experiments. *p<0.05, **p<0.01, ***p<0.001, ****p<0.0001 vs. 0 mM metformin at the same glucose concentration unless indicated otherwise, as determined by two-way ANOVA with Dunnett’s or Šidak’s *post-hoc* test. Statistically significant interaction between metformin and glucose was confirmed by two-way ANOVA only for CD69 expression **(A)**.

To further evaluate how the alterations in cellular energetics affect the function of Jurkat cells, we next measured the effect of metformin treatment and glucose concentration on the activation of Jurkat cells (**Figure 3A**; **Supplementary Figure 3A**). 5 mM metformin significantly reduced CD69 expression at 25 mM and 5.6 mM glucose to about 80% of control level, with an even stronger effect at 0 mM glucose. On the other hand, 0.3 mM metformin did not significantly reduce CD69 expression regardless of glucose concentration. While high glucose level had no effect on the activation, low glucose level itself slightly reduced CD69 expression and potentiated the suppressive effect of metformin as confirmed by significant statistical interaction between metformin and glucose level. Jurkat cell activation was unaffected by insulin level regardless of concurrent metformin treatment (**Supplementary Figure 4**).

Interestingly, despite the reduction in activation, cytokine secretion was in large part preserved by metformin treatment (**Figures 3B, C**; **Supplementary Figure 3**) at normal or high glucose levels. There was a dose-dependent trend towards lower IL-2 secretion (not significant, **Figure 3B**) but no clear effect on IFN-γ secretion even with 5 mM metformin (**Figure 3C**) was observed. Instead, IFN-γ secretion was more dependent on the glucose levels, with significantly lower levels in 0 mM versus 5.6 mM glucose. The same trend for low glucose was also observed in 5 mM metformin treated cells, although interestingly, IFN-γ secretion was significantly lower in 25 mM versus 5.6 mM glucose in this case. On the other hand, IL-2 secretion was unaffected by low glucose itself. Although the effect of 5 mM metformin on IL-2 secretion appeared stronger at 0 mM glucose, no significant interaction between metformin and glucose level could be confirmed for either cytokine. Overall, metformin treatment at normal glucose levels only partially suppressed Jurkat cell activation and partly preserved cytokine secretion, while metformin treatment at low glucose led to a stronger suppression of Jurkat cell activation and possibly cytokine secretion.

In order to investigate whether these effects were mediated by AMPK activation, we performed the experiment in the presence of AMPK inhibitor compound C. We found that blocking AMPK returned CD69 expression in metformin treated cells in low glucose back to the control levels (**Figure 3D**). However, compound C itself strongly suppressed IL-2 secretion regardless of metformin treatment (**Figure 3E**). Direct AMPK activator A769662 and mTOR inhibitor rapamycin both strongly reduced IL-2 secretion as well. Overall, while AMPK activation likely plays a role in reducing CD69 expression and possibly IL-2 secretion by metformin in low glucose, the latter could not be unequivocally confirmed.

### 3.4 Metformin reduces PD-1 expression but does not improve mitochondrial mass in Jurkat cells

**Figure 4:**
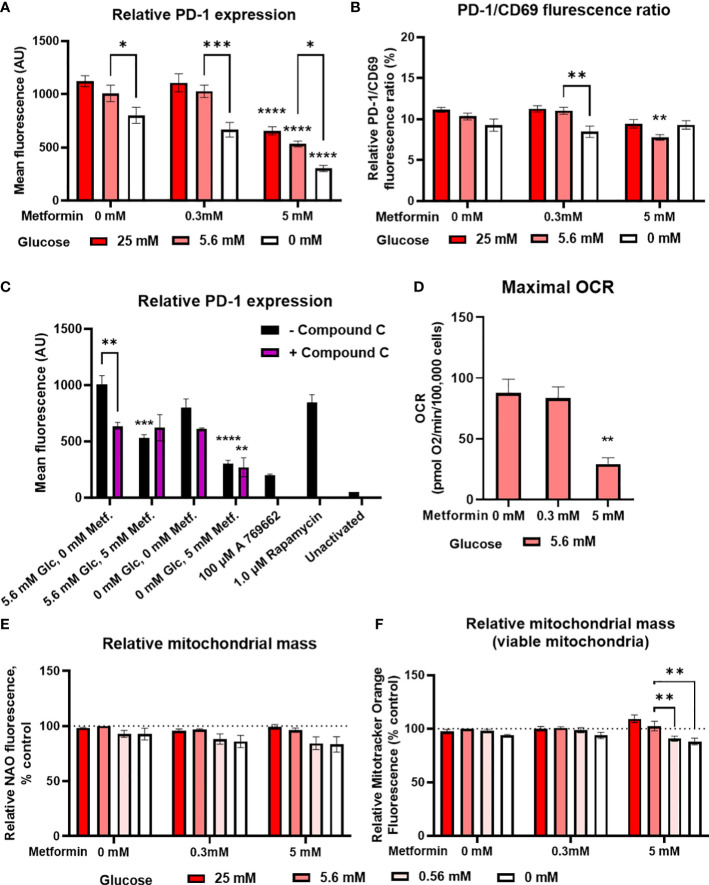
The effect of metformin as a function of glucose availability on markers of Jurkat cell exhaustion and metabolic fitness. Jurkat cells were grown in media with different glucose (denoted by shades of red) and metformin concentrations for 24 h **(A–C)**, 48 h **(D)** or 72 h **(C)**. **(A–C)** The cells were activated with anti-CD3/anti-CD28 antibodies and relative surface expression of PD-1 and CD69 **(B)** was measured by flow cytometry. The ratio of PD-1/CD69 fluorescence was calculated **(B)**. In **(C)**, Jurkat cells were pretreated with 5μM compound C (AMPK inhibitor) for 30 min, followed by metformin treatment at 5.6 mM or 0.56 mM glucose and anti-CD3/anti-CD28 activation for 24h. **(D)** Maximal respiratory capacity was determined by Seahorse Mito Stress Assay, measured as maximal OCR after the injection of 1.5 μM FCCP. **(E, F)** Relative mitochondrial mass was determined by NAO **(E)** or Mitotracker Orange **(F)** staining and flow cytometry. The mean ± SEM is shown for three **(A–C, E, F)** or five **(D)** independent experiments. *p<0.05, **p<0.01, ***p<0.001, ****p<0.0001 vs. 0 mM metformin at the same glucose concentration unless indicated otherwise, as determined by two-way ANOVA **(A-C, E, F)** or one-way ANOVA **(D)** with Dunnett’s *post-hoc* test. No significant interaction between metformin and glucose level could be confirmed by two-way ANOVA **(A, B, E, F)**.

Since recent studies have shown that the anti-tumor effects of metformin in animal models are at least partially mediated by modulation of the immune system (10–12, 66) and that metformin can to some extent improve the therapy with immune checkpoint inhibitors (18–21, 67), we next investigated whether metformin treatment could improve Jurkat cell exhaustion and metabolic fitness. We determined PD-1 expression and mitochondrial mass as the main parameters of metabolic fitness. PD-1 expression on activated Jurkat cells was significantly reduced by 5 mM but not 0.3 mM metformin treatment (**Figure 4A**; **Supplementary Figure 3E**) regardless of glucose. PD-1 levels were significantly lower in 0 mM versus 5.6 mM glucose. Though there was a weak but consistent trend towards higher PD-1 level in high glucose, neither high glucose nor insulin levels themselves significantly affected PD-1 expression, and insulin did not modify the effect of metformin (**Supplementary Figure 4**). Additionally, no significant statistical interaction between metformin and glucose level could be confirmed. Most of the observed effects of metformin and low glucose were likely caused by the reduced activation, as PD-1/CD69 ratio was only slightly reduced by metformin treatment or by low glucose level itself (**Figure 4B**). The effect of metformin on the reduced PD-1 expression was likely independent of AMPK, as blocking AMPK activation did not normalize PD-1 expression in metformin treated cells (**Figure 4C**).

In terms of mitochondrial function, metformin treatment did not increase either the total (**Figure 4E**) or viable (i.e. not depolarized, **Figure 4F**) mitochondrial mass in Jurkat cells. Low glucose even showed a trend towards lower mitochondrial mass. There was also no increase in maximal respiratory capacity, which was actually significantly decreased by 5 mM metformin treatment (**Figure 4D**). Taken together, metformin treatment does not appear to improve the Jurkat cell metabolic fitness directly, regardless of glucose availability.

### 3.5 2-deoxy-D-glucose stimulates IFN-γ secretion and improves markers of exhaustion in Jurkat cells at a physiologically achievable concentration

**Figure 5:**
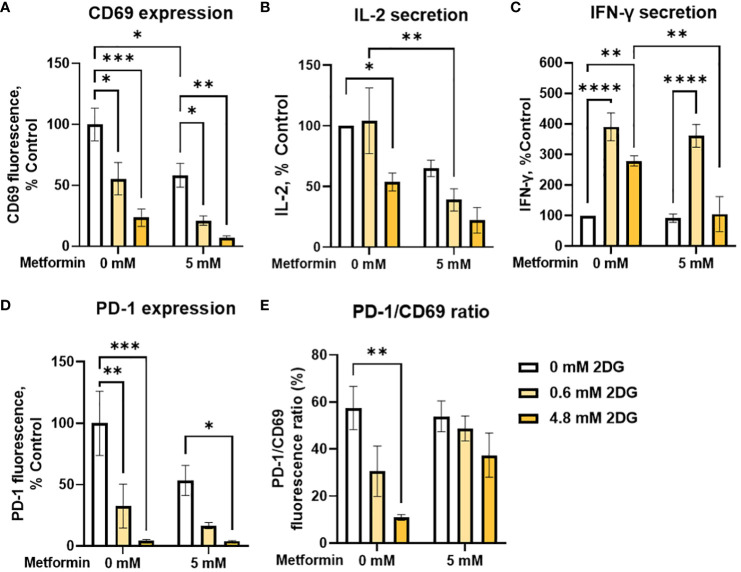
The effect of metformin and 2-deoxy-D-glucose treatment on Jurkat cell activation, exhaustion and effector functions. Jurkat cells were activated with PMA/ionomycin and treated with metformin and/or 2DG as indicated in medium supplemented with 5.6 mM glucose for 24h (data adapted from (60) according to Creative Commons Attribution license (http://creativecommons.org/licenses/by/4.0/). **(A, D, E)** Relative surface expression of CD69 **(A, E)** and PD-1 **(D, E)** was determined by flow cytometry. The concentration of IL-2 **(B)** and IFN-γ **(C)** in the medium was determined by ELISA. The mean ± SEM is shown for three **(A, D, E)** or four **(B, C)** independent experiments. *p<0.05, **p<0.01. ***p<0.001, ****p<0.0001 vs. untreated control unless indicated otherwise, as determined by two-way ANOVA with Dunnett's or Šidak's *post-hoc* test. No significant interaction between metformin and 2DG could be confirmed by two-way ANOVA.

In addition to metformin, 2DG has been shown to have specific effects on protein N-glycosylation that affect the expression of both PD-1 and PD-L1 (57, 58, 60, 62), suggesting its potential as an adjuvant to other immunomodulatory treatments. We therefore tested 2DG and its combination with metformin for the effects on T cell functionality and exhaustion markers. 2DG significantly reduced CD69 expression in activated Jurkat cells in a dose-dependent manner (**Figure 5A**). Low, physiologically achievable 2DG concentration (0.6 mM) caused about a 50% drop in CD69 levels, while higher (4.8 mM) 2DG concentration reduced them to about 20% of control levels. The concurrent treatment with metformin further reduced CD69 expression, but synergism could not be confirmed by two-way ANOVA. IL-2 secretion was unaffected by 0.6 mM 2DG and reduced to about 50% of control by 4.8 mM 2DG (**Figure 5B**). IL-2 levels were further reduced in the presence of metformin for both 2DG concentrations with metformin significantly reducing IL-2 levels in the presence of 0.6 mM 2DG. Interestingly, 0.6 mM 2DG treatment led to an almost 300% increase in IFN-γ levels (**Figure 5C**). This effect was also present in combination with metformin treatment and even in low glucose conditions (**Supplementary Figure 5**). The effect of 4.8 mM 2DG was slightly less pronounced and abrogated by concurrent treatment with metformin. Nevertheless, statistical interaction between metformin and 2DG could not be confirmed.

We next investigated the effect of 2DG and metformin on PD-1 expression in activated Jurkat cells. We observed a dose-dependent decrease in surface PD-1 with 2DG, with a strong (~70%) reduction even at low, 0.6 mM 2DG, whereas 4.8 mM 2DG almost completely suppressed PD-1 expression (**Figure 5D**). This effect was also observed in the presence of metformin, although there was no additional effect on the reduction in PD-1 levels over 2DG alone. Importantly, 2DG treatment decreased the PD-1/CD69 ratio in a dose-dependent manner (significant at 4.8 mM 2DG), indicating that the effect of 2DG on PD-1 expression was stronger than that on Jurkat cell activation (**Figure 5E**). This was less apparent in combination with metformin, possibly due to already very low PD-1 levels with 2DG alone. Overall, these results therefore indicate that 2DG at physiologically relevant concentrations (0.6mM) significantly increases IFN-γ secretion, and reduces PD-1 more strongly than CD69.

### 3.6 Metformin induces a shift in differentiation status towards effector subsets in T cells from PBMC

**Figure 6:**
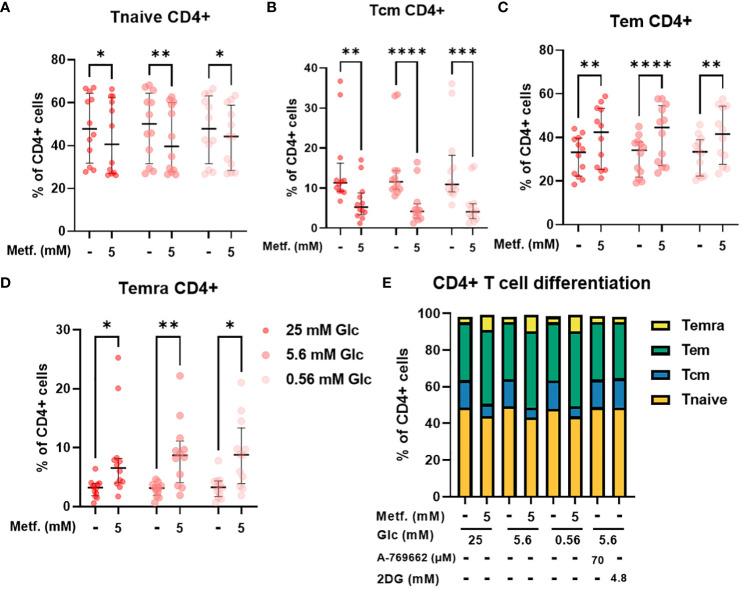
The effect of metformin as a function of glucose availability on CD4+ T cell differentiation in PBMC. PBMC were treated for 72h with metformin in media supplemented with 25 mM, 5.6 mM or 0.56 mM glucose (denoted by shades of red). AMPK activator A 769662 and 4.8 mM 2DG were used as additional controls. After treatment, the cells were stained with the appropriate antibodies and analyzed by flow cytometry. The CD3+ CD4+ population was divided into four populations according to CD45RA and CCR7 (CD197) expression: naïve T cells (Tnaive, CD45RA+ CCR7+, **(A)**), central memory T cells (Tcm, CD45RA- CCR7+, **(B)**), effector memory T cells (Tem, CD45RA- CCR7-, **(C)**) and terminally differentiated effector T cells (Temra, CD45RA+ CCR7-, **(D)**). The gating strategy is shown in section 1.2.4 of the **Supplementary Material**. The percentage of total CD4+ CD3+ cells is shown for each subpopulation. The data points represent individual experiments, while the horizontal lines and error bars represent the median percentage ± interquartile range. Three independent experiments were performed for each of the four healthy donors for a total of twelve independent experiments. *p<0.05, **p<0.01, ***p<0.001, ****p<0.0001 as determined by repeated measures two-way ANOVA with Dunnett (glucose) or Šidak (metformin) *post-hoc* test. No significant interaction between metformin and glucose level could be confirmed by repeated measures two-way ANOVA. The distribution of subpopulations according to differentiation status is summarized in **(E)** by displaying mean percentages of the subsets.

We next used PBMC from healthy donors to validate our findings in Jurkat cells on a more physiologically relevant T cell model. PBMC comprise several cell types (mainly monocytes, B cells and T cells) with considerable heterogeneity. One important source of this heterogeneity is the differentiation of T cells into naïve, central memory, effector memory and terminally differentiated effector subsets. Since these subsets display different effector function and proliferation potential, the balance between these differentiation subsets is an important determinant of a successful anti-tumor immune response (68, 69). We therefore studied the effect of metformin on differentiation status of T cells from PBMC as a function of glucose concentration. While 0.3 mM metformin did not affect T cell differentiation (**Supplementary Figure 6**), we found that 5 mM metformin significantly reduced the percentage of naïve T cells within CD4+ T cells, although the effect was rather small (5-10% lower median percentage) and the scatter considerable (**Figure 6A**). The percentage of central memory T cells was also reduced by about 10% with 5 mM metformin treatment, with an even clearer trend (**Figure 6B**). Conversely, the percentage of both of the effector subsets (effector memory and terminally differentiated effector cells) were significantly increased by 5 mM metformin treatment by about 5-10% in each case (**Figures 6C, D**), suggesting an overall shift to effector subsets with metformin treatment. This effect was unaffected by glucose levels, as neither glucose level nor its interaction with metformin had any significant effect. CD4+ differentiation was also unaffected by AMPK activator A 769662 or 2DG treatment (**Figure 6E**), suggesting that the effect of metformin was specific to its metabolic effect rather than a general induction of energy stress.

**Figure 7:**
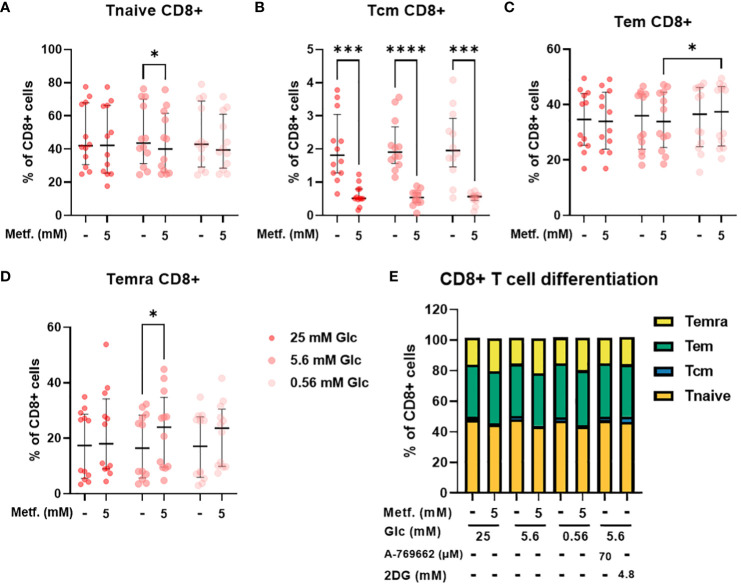
The effect of metformin as a function of glucose availability on CD8+ T cell differentiation in PBMC. PBMC were treated for 72h with metformin in media supplemented with 25 mM, 5.6 mM or 0.56 mM glucose (denoted by shades of red). AMPK activator A 769662 and 4.8 mM 2DG were used as additional controls. After treatment, the cells were stained with the appropriate antibodies and analyzed by flow cytometry. The CD3+ CD8+ population was divided into four populations according to CD45RA and CCR7 (CD197) expression: naïve T cells (Tnaive, CD45RA+ CCR7+, **(A)**), central memory T cells (Tcm, CD45RA- CCR7+, **(B)**), effector memory T cells (Tem, CD45RA- CCR7-, **(C)**) and terminally differentiated effector T cells (Temra, CD45RA+ CCR7-, **(D)**). The gating strategy is shown in section 1.2.4 of the **Supplementary Material**. The percentage of total CD8+ CD3+ cells is shown for each subpopulation. The data points represent individual experiments, while the horizontal lines and error bars represent the median percentage ± interquartile range. Three independent experiments were performed for each of the four healthy donors for a total of twelve independent experiments. *p<0.05, ***p<0.001, ****p<0.0001 as determined by repeated measures two-way ANOVA with Dunnett (glucose) or Šidak (metformin) *post-hoc* test. No significant interaction between metformin and glucose level could be confirmed by repeated measures two-way ANOVA. The distribution of subpopulations according to differentiation status is summarized in **(E)** by displaying mean percentages of the subsets.

In CD8+ T cells, the percentage of central memory T cells was reduced regardless of glucose, with the same effect in relative terms as in CD4+ cells, but the absolute effect size was smaller due to a much smaller Tcm CD8+ population (**Figure 7B**). The percentage of naïve CD8+ T cells was also only significantly reduced by 5 mM metformin at 5.6 mM glucose with a similar trend at 0.56 mM glucose (**Figure 7A**). Conversely, the percentage of terminally differentiated effector CD8+ T cells was also only significantly increased by 5 mM metformin at 5.6 mM with a similar trend at other two glucose concentrations (**Figure 7D**). Interestingly, metformin had no clear effect on the effector memory CD8+ T cell population (**Figure 7C**). Similarly to CD4+ T cells, CD8+ differentiation was mostly unaffected by glucose level, A 769662 or 2DG treatment (**Figure 7E**). The only exception was the higher percentage of Tem CD8+ cells in metformin treated cells at 0.56 mM as opposed to 5.6 mM glucose (**Figure 7C**). There was also no significant interaction between metformin and glucose level. Overall, the effect of metformin on CD8+ T cell differentiation was qualitatively similar to CD4+ T cells but less pronounced with no clear effect on the effector memory subset.

### 3.7 Metformin induces a shift towards glycolysis in resting T cells and restricts oxidative ATP production upregulation in activated T cells from PBMC

**Figure 8:**
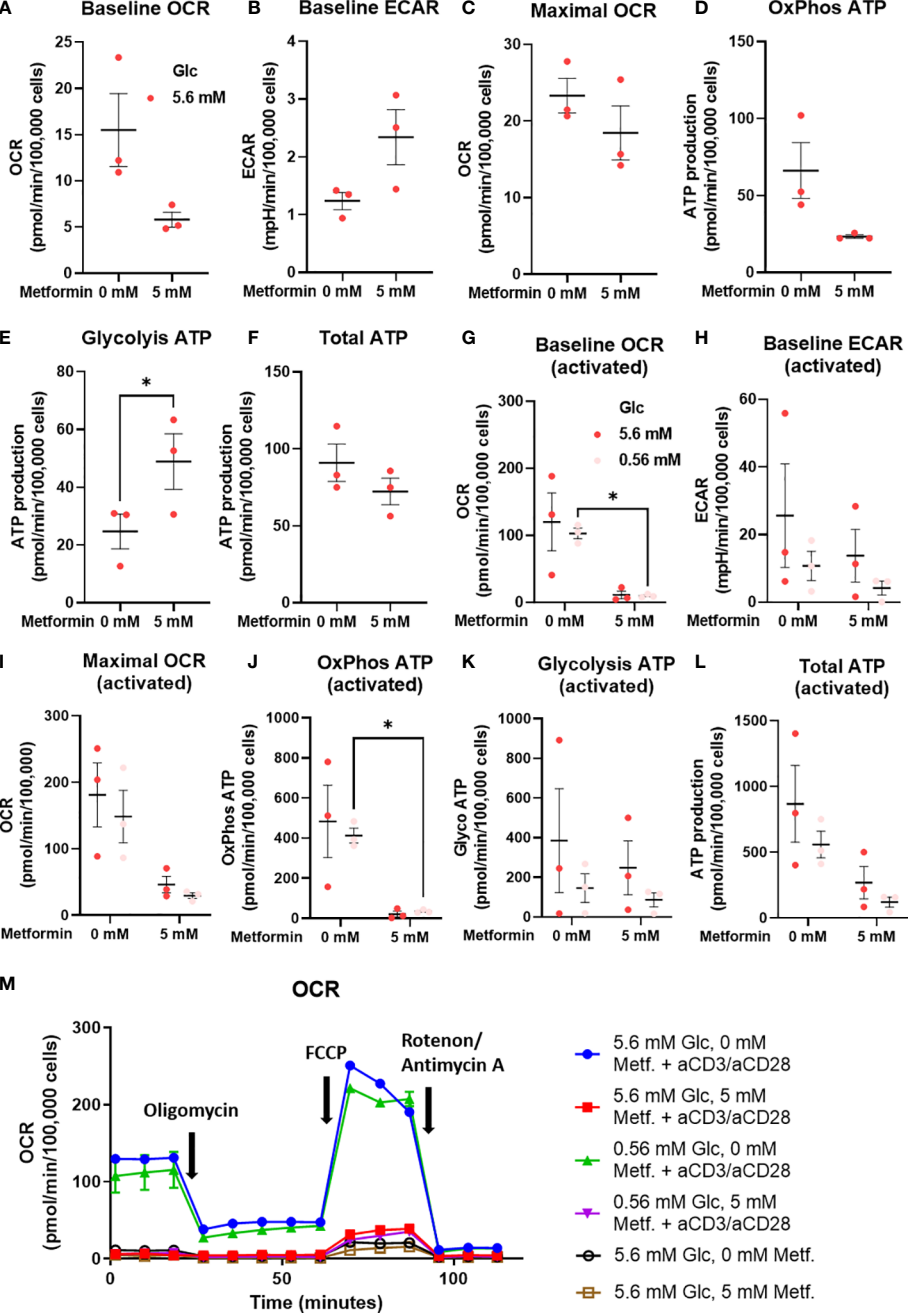
The effect of the metformin treatment on energy metabolism of T cells from PBMC. **(A–F)** PBMC cells were seeded in media with 5.6 mM glucose and treated for 48h with 0 mM or 5 mM metformin as indicated. **(G–L)** T cells from PBMC were activated with anti-CD3/anti-CD28 antibodies and treated with 0 mM or 5 mM metformin in media with 5.6 mM or 0.56 mM glucose for 48h as indicated. Baseline OCR **(A, G)** and ECAR **(C, H)** as well as maximal OCR **(C, I)** were determined using Seahorse Mito Stress Test assay with 1.5 μM oligomicin, 2 μM FCCP and 0.5 μM rotenone/antimycin A injections. The ATP production from oxidative phosphorylation **(D, J)** and glycolysis **(E, K)**, as well as total ATP production **(F, L)** were calculated according to the manufacturer’s instructions for Seahorse Real Time ATP Assay. Mean ± SEM is shown for three independent experiments with different donors. *p<0.05 as determined by paired t-test **(A–F)** or two-way ANOVA with Šidak *post-hoc* test **(G–L)**. A representative time-course for OCR is shown in **(M)**.

As the effects on T cell differentiation appeared to be more dependent on the specific metabolic effect of metformin that general energy stress, we next measured the cellular energy metabolism of metformin treated T cells from PBMC using the Seahorse analyzer. We found a clear trend towards lower baseline OCR and ATP production from oxidative phosphorylation (**Figures 8A, D**) with metformin treatment. Conversely, ECAR (not significant) and glycolytic ATP production (**Figures 8B, F**) were increased, allowing full compensation of total ATP production (**Figure 8G**). Despite this shift, maximal respiratory capacity appeared to be unaffected (**Figure 8C**). Overall, metformin induced a clear shift from oxidative to glycolytic ATP production without reducing total ATP production.

To explore whether the same effect was present in activated metformin treated T cells from PBMC, we also measured their metabolism at both normal and low glucose levels. We found that 5 mM metformin almost completely suppressed both baseline OCR and OxPhos ATP production (**Figures 8G, J**). This effect was present at both 5.6 mM and 0.56 mM glucose, although only statistically significant at latter. Maximal respiratory capacity was also reduced (**Figure 8I**) to a lesser extent (not significant). Interestingly, no compensatory increase in ECAR or glycolytic ATP production was observed at either glucose level (**Figures 8H, J**). This led to a substantial (though not significant) reduction in total ATP production in 5 mM metformin treated cells, particularly in low glucose where glycolysis is restricted by glucose availability (**Figure 8L**). Taken together, the results suggest metformin treatment at higher concentrations can restrict the upregulation of total ATP generation in activated T cells.

### 3.8 Metformin treatment improves the balance between the activation and exhaustion markers in T cells from PBMC

**Figure 9:**
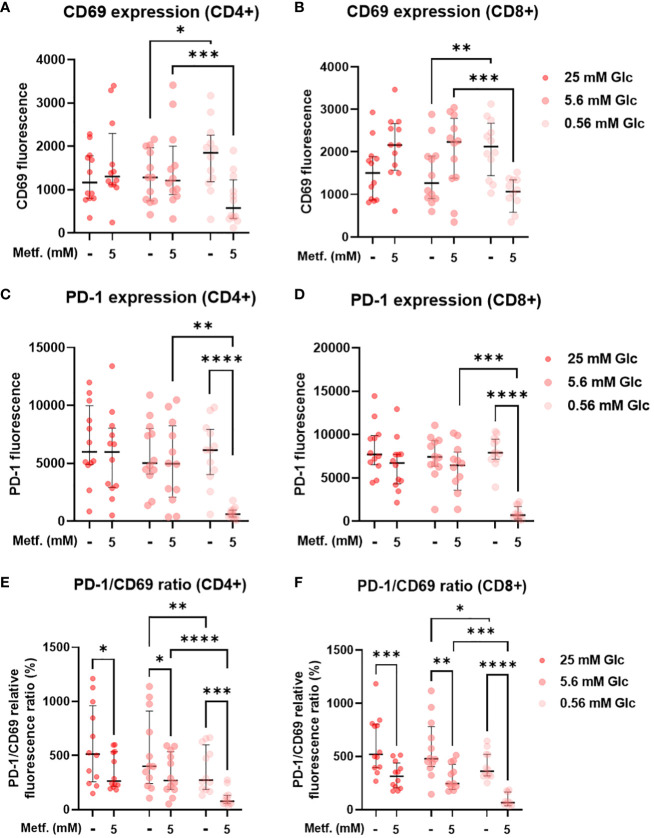
The effect of metformin as a function of glucose availability on T cell activation and PD-1 expression in activated T cells from PBMC. PBMC were treated for 72h with metformin in media supplemented with 25 mM, 5.6 mM or 0.56 mM glucose (denoted by shades of red) and activated with anti-CD3 and anti-CD28 antibodies during the treatment. After treatment, the cells were stained with antibodies and analyzed by flow cytometry. The expression levels of activation marker CD69 **(A, B)**, exhaustion marker/immune checkpoint PD-1 **(C, D)** and the ratio of PD-1/CD69 fluorescence **(E, F)** were determined in CD4+ **(A, C, E)** and CD8+ **(B, D, F)** T cells. The gating strategy is shown in section 1.2.5 of the **Supplementary Material**. The data points represent individual experiments, while the horizontal lines and error bars represent the median percentage ± interquartile range. Three independent experiments were performed for each of the four healthy donors for a total of twelve independent experiments. *p<0.05, **p<0.01, ***p<0.001, ****p<0.0001 as determined by repeated measures two-way ANOVA with Dunnett (glucose) or Šidak (metformin) *post-hoc* test. Repeated measures two-way ANOVA confirmed the significant interaction between metformin and glucose level for CD69 **(A, B)** and PD-1 **(C, D)** expression, but not the PD-1/CD69 ratio **(E, F)**.

To explore the effect of metformin on primary T cell activation as a function of glucose availability, we activated T cells in PBMC with anti-CD3/anti-CD28 antibodies during metformin treatment and measured the expression of the activation marker CD69. We found that neither 0.3 mM (**Supplementary Figures 7A, B**) nor 5 mM metformin alone had any significant effect on CD69 expression in either CD4+ or CD8+ T cells (**Figures 9A, B**). In both of these subsets, a trend towards lower CD69 expression was observable with metformin treatment at 0.56 mM glucose. In case of CD8+ T cells, a weak opposite trend could be observed for 5.6 mM and 25 mM glucose. There was significant statistical interaction between metformin and glucose level in both CD4+ and CD8+ T cells, with 0.56 mM glucose significantly increasing CD69 expression in the absence of metformin and significantly reducing it in 5 mM metformin treated cells. Both metformin and glucose individually, as well as their interaction also significantly affected the expression of PD-1 in both T cell subsets. While 0.3 mM metformin treatment (**Supplementary Figures 7C, D**), or 5 mM metformin treatment at 5.6 mM or 25 mM glucose had no clear effect, 5 mM metformin significantly reduced PD-1 levels at 0.56 mM glucose (**Figures 9C, D**). The same was true for the effect of low glucose, which only significantly reduced PD-1 expression in the presence of metformin. On the other hand, blocking glycolysis by 2DG was sufficient to suppress both CD69 and PD-1 expression (**Supplementary Figure 8**). To differentiate the effect on PD-1 expression from that on activation, we also calculated the PD-1/CD69 fluorescence ratio, which was significantly reduced by 5 mM metformin regardless of glucose concentration in both CD4+ and CD8+ T cells (**Figures 9E, F**) but unaffected by 0.3 mM metformin (**Supplementary Figures 7E, F**). Two-way ANOVA could not confirm the interaction between metformin and glucose level. However, 0.56 mM glucose itself also significantly reduced the PD-1/CD69 ratio regardless of metformin. Overall, high metformin improved the balance between the activation and exhaustion markers in both the activated CD4+ as well as in the activated CD8+ T cells from PBMC and did not markedly reduce T cell activation as long as sufficient glucose was present.

### 3.9 Metformin partially reduces proliferation and cytokine secretion in T cells from PBMC

**Figure 10:**
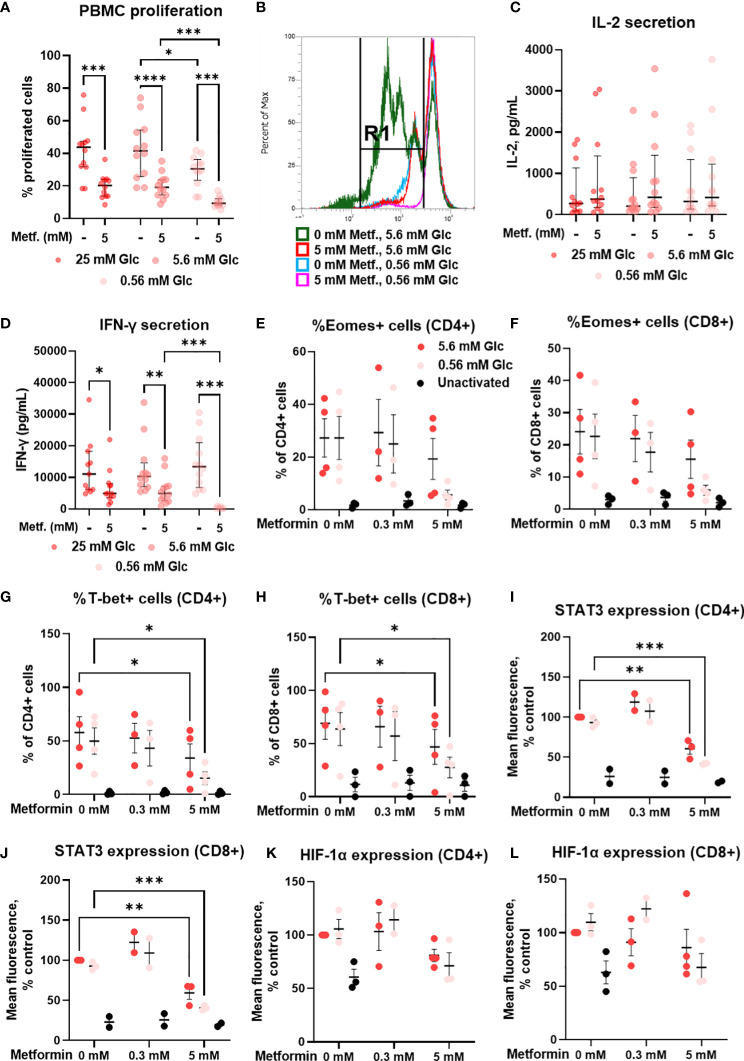
The effect of metformin as a function of glucose availability on proliferation, cytokine secretion and transcription factors in activated T cells from PBMC. PBMC were treated for 72h **(A–D)** or 48h **(E–L)** with 0.3 mM or 5 mM metformin as indicated in media supplemented with 25 mM, 5.6 mM or 0.56 mM glucose (denoted by shades of red) and activated with anti-CD3 and anti-CD28 antibodies during the treatment. **(A, B)** The percentage of proliferated PBMC cells following T cells activation was determined by measuring CFSE dilution by flow cytometry. A representative histogram of CFSE fluorescence is shown in **(B)**. **(C, D)** The concentration of secreted IL-2 after 24h treatment **(C)** and IFN-γ in after 72h treatment **(D)** in culture media was determined with ELISA. **(E–L)** The expression of Eomes **(E, F)**, T-bet **(G, H)**, STAT3 **(I, J)** and HIF-1α **(K, L)** was measured by intracellular staining flow cytometry. The gating strategy is shown in section 1.2.6 of the **Supplementary Material**. **(A–D)** The data points represent individual experiments, while the horizontal lines and error bars represent the median percentage ± interquartile range. Three independent experiments were performed for each of the four healthy donors for a total of twelve independent experiments. *p<0.05, **p<0.01, ***p<0.001, ****p<0.0001 as determined by repeated measures two-way ANOVA with Dunnett (glucose) or Šidak (metformin) *post-hoc* test. Repeated measures two-way ANOVA confirmed the significant interaction between metformin and glucose level for IFN-γ secretion only **(D)**. **(E–L)** The data points represent individual experiments, while the horizontal lines and error bars represent the mean ± SEM. One independent experiment was performed for each of three to four donors. *p<0.05, ***p<0.001, as determined by mixed effects model **(E–H)** or two-way ANOVA **(I–L)** with Dunnett (metformin) or Šidak (glucose) *post-hoc* test.

We next analyzed the effects of metformin on the proliferation capacity and on the secretion of the effector cytokines of T cells, which are key determinants of a successful anti-tumor immune response. 5 mM metformin treatment significantly reduced the percentage of proliferated PBMC cells from about 40% to about 20% at 25 mM and 5.6 mM glucose, with a similar effect observed at 0.56 mM glucose (**Figures 10A, B**). Low glucose concentration itself also significantly reduced the percentage of proliferated cells by about 10%. An even stronger effect was observed for 2DG treatment (**Supplementary Figure 9**). There was, however, no significant interaction between metformin and glucose level.

In parallel, we investigated the secretion of IL-2 and IFN-γ, two key cytokines for anti-tumor immunity. Metformin treatment had no significant effect on IL-2 secretion after 24h, although a weak trend may be noted towards higher IL-2 levels at both 0.3 mM and 5 mM (**Figure 10C**; **Supplementary Figure 7G**). There was also no clear effect of glucose concentration or glycolysis inhibition by 4.8 mM 2DG on IL-2 secretion (**Supplementary Figure 9**). In contrast, IFN-γ levels were unaffected by 0.3 mM metformin (**Supplementary Figure 7H**) and significantly reduced by 5 mM metformin treatment at all glucose concentrations (**Figure 10D**). The effect of metformin was significantly potentiated by low glucose levels as confirmed by the significant interaction between metformin and glucose, and the significant reduction in IFN-γ secretion in metformin treated cells at 0.56 mM versus 5.6 mM glucose. Although IFN-γ secretion was not reduced in low glucose in the absence of metformin, 4.8 mM 2DG did significantly suppress IFN-γ secretion (**Supplementary Figure 9**). Overall, while metformin only partially reduced T cell proliferation and cytokine secretion when sufficient glucose was available, the suppression of T cell function was a lot more potent at low glucose concentration.

### 3.10 Metformin at 5 mM concentration restricts the upregulation of key T cell transcription factors in T cells from PBMC

To explore the mechanism behind reduced IFN-γ secretion, we next measured the expression of some key transcription factors involved in T cell activation and effector functions. We found that 5 mM metformin treatment reduced the expression of T-bet and STAT3 in both CD4+ and CD8+ T cells (**Figures 10G-J**). While already present at normal glucose, this effect was especially pronounced in low glucose conditions, though no statistical interaction between the metformin and the glucose level could be confirmed. The same trend was observed for Eomes (**Figures 10E, F**) and to a lesser extent HIF-1α expression (**Figures 10K, L**). In most cases, the expression levels of these transcription factors were still higher compared to unactivated T cells, in line with the effect of metformin on cytokine secretion. 0.3 mM metformin did not reduce the expression of these transcription factors and even exhibited an opposite trend in some cases.

**Figure 11:**
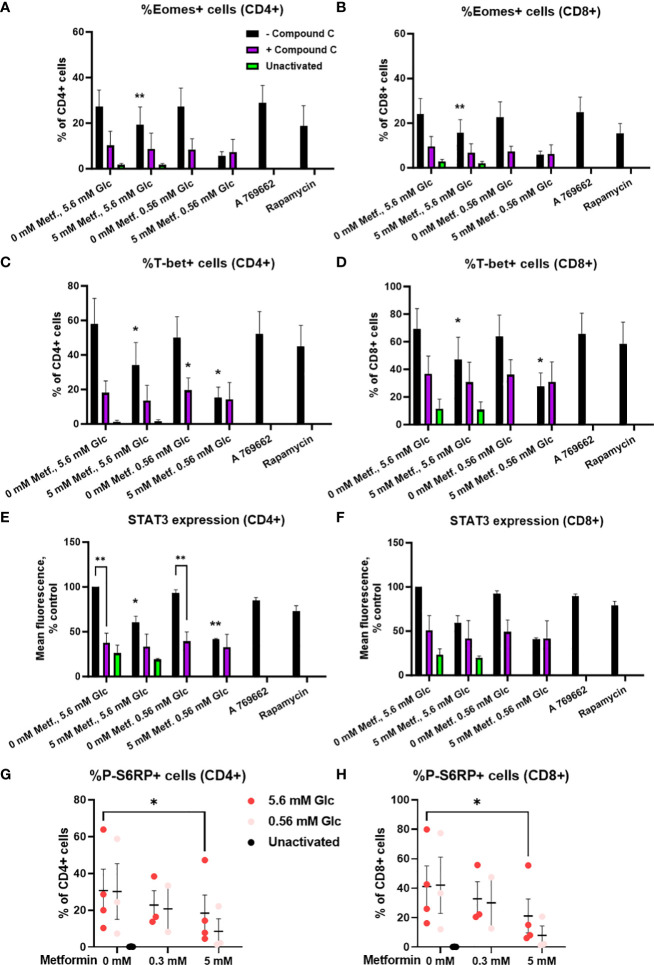
The effect of metformin as a function of glucose availability on mTOR signaling and the role of AMPK in activated T cells from PBMC. **(A–F)** PBMC were pretreated with 5 μM compound C for 30 min and subsequently treated for 48h with 5 mM metformin as indicated in media supplemented with 5.6 mM or 0.56 mM glucose and activated with anti-CD3 and anti-CD28 antibodies during the treatment. The expression of Eomes **(A, B)**, T-bet **(C, D)** and STAT3 **(E, F)** was measured by intracellular staining flow cytometry. **(G, H)** PBMC were treated for 48h with 0.3 mM or 5 mM metformin as indicated in media supplemented with 5.6 mM or 0.56 mM glucose (denoted by shades of red) and activated with anti-CD3 and anti-CD28 antibodies during the treatment. The expression of phosphorylated S6RP was measured by intracellular staining flow cytometry. The gating strategy is shown in section 1.2.6 of the **Supplementary Material**. The data points represent individual experiments, while the horizontal lines and error bars represent the mean percentage ± SEM. One independent experiment was performed for each of three to four donors. *p<0.05, **p<0.01, as determined by mixed effects model **(A–D, G, H)** or two-way ANOVA **(E, F)** with Dunnett or Šidak *post-hoc* test.

We next investigated whether these effects were AMPK-dependent by measuring transcription factor expression in the presence of compound C. We found that the expression of these transcription factors was strongly reduced by compound C to a level similar to 5 mM metformin treatment in low glucose (**Figures 11A-F**; **Supplementary Figure 10**). Conversely, direct AMPK activation by A769662 did not markedly reduce their expression, suggesting that AMPK activation is probably not the primary mechanism responsible for the effects of metformin. Another signaling pathway related to the energy status is the mTOR pathway involved in cellular growth and proliferation. Metformin at 5 mM significantly reduced the phosphorylation of S6 ribosomal protein (S6RP), its downstream target (**Figures 11G, H**). This effect appeared even stronger in low glucose, though it was not statistically significant. However, while rapamycin reduced S6RP phosphorylation to a similar extent to metformin (particularly in low glucose), it only slightly reduced the expression of Eomes and STAT3, suggesting that while mTOR suppression is likely involved in the effects of metformin, additional mechanism linked to its direct metabolic effect are also required for its full effect.

## 4 Discussion

In the present study, we investigated the effect of metformin as a function of glucose levels in Jurkat cells and T cells from PBMC as two T cell models. We primarily focused on the effector functions and markers of exhaustion and metabolic fitness of T cells. In parallel, we investigated the effects of another metabolic inhibitor − 2-deoxy-D-glucose (2DG) on the potential improvement of the markers of exhaustion and activation of Jurkat cells.

Glucose has been shown to be a key nutrient determining the response to metformin in cancer cells (9, 35) as well as being involved in regulating T cell function (45, 52). A very recent report has also pointed to the role glucose availability might play in mouse CD8+ T cell response to metformin (25). While other nutrients can importantly impact the T cell function both directly (44, 69) and indirectly (70–72), our results confirmed the key role of the glucose as opposed to the glutamine (**Figure 1**) or pyruvate (results not shown) in survival and proliferation of Jurkat cells used as a T cell model. Moreover, glucose levels can be highly variable in cancer patients, ranging from practically zero in the tumor core to significantly beyond 11 mM in the plasma of untreated or unsuccessfully treated diabetes patients, as well as those also receiving additional glucocorticoid treatment (47, 48). We therefore systematically studied the effect of metformin on T cells at different glucose concentrations. 5.6 mM glucose was used to simulate the normal physiological glucose level in the plasma of healthy people. To represent the high plasma glucose concentrations that can also be achieved in the clinical setting, 25 mM glucose was used in our 72 h experiments to simulate the long term exposure to more mildly elevated glucose levels experienced by T cells *in vivo* (around 11 mM). Moreover, 25 mM glucose is routinely used in high glucose cell culture media *in vitro*, so understanding the effects of metformin at such concentrations compared to more physiological glucose levels could help us better interpret the results of previous *in vitro* studies. The 0.56 mM and/or 0 mM glucose levels were used to simulate conditions in the tumor microenvironment, with the latter representing the extremely nutrient-deprived tumor core. We have to stress that some small amount of glucose was always present in the medium due to serum supplementation (<0.7 mM). In case of PBMC, this could potentially introduce a source of variability due to the use of autologous sera, so in that model we focused on media supplemented with 0.56 mM glucose as the low glucose condition.

### 4.1 The effect of metformin on T cells at normal physiological glucose levels

We found that at physiological levels of glucose, metformin concentrations achievable *in vivo* (0.03 mM to 0.3 mM) had very little to no suppressive effect on Jurkat cells in terms of their survival, proliferation, activation or effector functions. Similarly, no suppressive effects were observed for 0.3 mM metformin treatment in T cells from PBMC. This is consistent with our previous study (60) and several recent reports indicating that low concentrations of metformin may actually directly improve the anti-tumor function of T cells, including their proliferation and IFN-γ secretion (23–26). At higher concentrations (1 mM and especially 5 mM) of metformin used to mimic potential accumulation with chronic exposure, metformin did significantly reduce Jurkat cell number without increasing the percentage of dead cells. Therefore, under these conditions, metformin mostly reduced proliferation rather than induced cell death (**Figure 1**). This is consistent with the observed effect on the Jurkat cell bioenergetics, where metformin treatment alone failed to induce a severe energy crisis despite the dose-dependent suppression of the oxidative ATP production by metformin (**Figure 2**). The total ATP production was unchanged by 0.3 mM metformin and only very modestly reduced by 5 mM metformin due to the low overall contribution of OxPhos to ATP production in Jurkat cells. This suggests that additional mechanisms such as changes in cell signaling downstream of a relatively mild reduction in ATP production or altered redox balance are likely responsible for the observed reduced proliferation. Indeed, we have previously observed an almost complete suppression of mTOR signaling in Jurkat cells with 5 mM metformin that was not present at 0.3 mM (60).

A similar effect on proliferation was found in the activated T cells from PBMC of healthy donors, where 5 mM metformin reduced the percentage of proliferated cells but no major increase in cell death was noted (data not shown). This lack of cell death is consistent with a previous study demonstrating increased resistance to apoptosis in T cells treated with low concentration of metformin *in vitro* and better T cell survival with metformin use *in vivo* (24, 55). As these studies used low concentrations of metformin, while others noted unchanged or slightly decreased proliferation of T cells with 2 mM (14) and decreased proliferation with 5 mM metformin (53), this in conjunction with our results indicates that the effect of metformin on T cell proliferation is strongly concentration-dependent.

Another important determinant of an effective T cell response is the differentiation status of T cells. In the context of adoptive T cell therapy, the less differentiated memory T cell subsets are more effective in clearing solid tumors due to their increased persistence and proliferation capacity following the adoptive transfer (68, 69). Metformin treatment induced a clear shift in CD4+ T cell differentiation from the naïve (Tn) and particularly central memory (Tcm) subsets to the effector memory (Tem) and terminally differentiated effector (Temra) T cells (**Figure 6**). A similar but less pronounced effect was observed in CD8+ T cells (**Figure 7**), where the Tcm percentage was clearly reduced by metformin, but the reduction in Tn and the corresponding increase in Temra was only significant at 5.6 mM glucose. The most likely reason for the latter was the much lower starting frequency of CD8+ as opposed to CD4+ Tcm cells. It is important to note that this effect was present in unactivated CD8+ T cells that have been reported to express only low levels of organic cation transporter 1 responsible for metformin entry into the cell (26). Our results therefore indicate that at least high concentrations of metformin can substantially affect T cells even without activation. The observed shift towards effector subsets was most likely caused by the metformin-induced suppression of respiration itself rather than a more general state of energy stress, as neither low glucose nor 4.8 mM 2DG treatment or direct AMPK activation by A 769662 recapitulated the same effect. This is consistent with the predominant role of the oxidative metabolism in Tn and Tcm cells, whereas the effector subsets are more reliant on glycolysis (73–76). Indeed, we observed a clear shift in ATP generation from oxidative phosphorylation to glycolysis in metformin treated PBMC cells (**Figure 8**), consistent with this notion. While it is not possible to unequivocally confirm from our data that this metabolic shift is the cause of the altered differentiation status, such an explanation is plausible given that suppression of glycolysis has been shown to increase the percentage of memory T cells in the context of adoptive T cell therapy (68). Overall, the observed shift in differentiation could indicate an increased capacity to engage effector functions, but could also limit the proliferation capacity and persistence of metformin treated cells following activation, since Tcm and memory-like T cells were shown to be superior in their *in vivo* persistence, proliferation and tumor clearing in the context of adoptive T cell therapy of cancer (68, 69, 77).

We observed a concentration-dependent effect of metformin on Jurkat cell activation, which was suppressed by 5 mM metformin to about 70% of control levels (**Figure 3**). This resulted in partially reduced IL-2 secretion while IFN-γ levels were mostly unaffected. This was even more true for the lower, physiologically more achievable metformin concentration. In the T cells from PBMC, activation as measured by CD69 expression was mostly unaffected by metformin regardless of concentration, and similarly the secretion of IL-2 measured at an early time point was not affected. On the other hand, IFN-γ secretion was significantly reduced by 5 mM but not 0.3 mM metformin treatment (to ~50% of control). Taken together, our results indicate that while T cell activation itself is not substantially impacted by the metformin treatment (especially in primary T cells), high but not low metformin concentrations can indeed partially suppress their effector function (particularly IFN-γ secretion) and proliferation *in vitro*.

This is consistent with increased proliferation and/or IFN-γ secretion (19, 23–26, 78) observed *in vivo* in mice models (19, 23, 26) or in patients (24, 78). Additionally, the *in vitro* experiments with lower metformin concentrations (0.3 mM or less) also did not reveal any suppressive effects on T cells (24–26), consistent with our results. When higher (e.g. 5 mM) concentrations were used, previous studies also reported a decrease in IFN-γ secretion (25) in agreement with our observations. Our results are also consistent with the emerging research demonstrating beneficial suppressive effects of metformin in the context of autoimmune disorders (27–33) or other instances of excessive immune activation (79). As high concentrations of metformin are often used to study the effects of metformin on cancer cells *in vitro*, it is therefore important to note that they can also have substantial suppressive effects on the T cell effector functions. On the other hand, the early steps of the anti-tumor T cell response, namely activation and secretion of IL-2, seem to be mostly preserved by metformin treatment, suggesting that the potential suppressive effects of metformin could be more relevant in modulating the effector stage rather than the initial antigen presentation, T cell activation and IL-2 induced expansion in the lymph nodes.

In this regards, it is important to note, that metformin treatment prevented the upregulation of oxidative phosphorylation seen in the activated T cells (**Figure 8**). In contrast to the resting (unactivated) PBMC cells, there was no compensatory increase in the glycolytic ATP production, which was similar to or lower than that of control cells, consistent with a slight reduction in HIF-1α expression with metformin treatment (**Figure 10**). This led to decreased total ATP production available for the energetically and biosynthetically demanding later events following the initial activation, such as proliferation (36) and cytokine secretion (80). This helps to explain the stronger effect of metformin on these processes than that on early activation measured by CD69 expression.

Mechanistically, 5 mM metformin treatment also reduced the expression of several transcription factors involved in T cell activation and cytokine secretion, particularly STAT3 and T-bet, the master transcription factor of IFN-γ producing Th1 cells. As the latter directly promotes the expression of IFN-γ in CD4+ T cells (81), while the former can also promote its secretion in some contexts (82), this could partially explain the reduction of its secretion with metformin treatment. The effect on the expression of Eomesodermin (Eomes) playing a similar role in IFN-γ secretion in CD8+ cells (83) was less pronounced, though a trend towards lower levels was observed as well. Low concentration of metformin (0.1 mM) has previously been shown to increase Eomes expression in CD8+ cells, leading to a reduction of PD-1 expression and an increased percentage of Tcm cells (24). While we did observe a similar weak trend with 0.3 mM metformin in unactivated T cells, no such increase was observed with 5 mM metformin. This, together with the trend towards reduced Eomes expression in 5 mM metformin treated activated T cells might also help to explain the observed reduction in Tcm populations (**Figures 6**, **7**). Overall, the results suggest that high metformin concentration can reduce the expression of key transcription factors involved in regulating IFN-γ transcription, which is in agreement with the observed reduction in its secretion.

AMPK and mTOR are two crucial signaling pathways linking the energy and nutrient status of the cell to other signaling pathways and subsequent cellular responses. Since metformin reduced the total ATP production in both Jurkat cells and activated T cells from PBMC, we hypothesized that AMPK activation could mediate the observed suppressive effects of metformin on their proliferation and cytokine secretion. To that end, we treated the model T cells with metformin in the presence of the AMPK inhibitor compound C and used the direct AMPK activator A 769662 as an additional control. While A 769662 did recapitulate some of the effects of metformin on Jurkat cells, it also induced considerable cell death (data not shown). It also did not affect the activation, differentiation or cytokine secretion in T cells from PBMC. Conversely, while compound C restored the CD69 expression in the metformin treated Jurkat cells, it did itself substantially block the secretion of IL-2 as well. Additionally, it also reduced the expression of T-bet, Eomes and STAT3 in primary T cells (**Figure 11**), possibly due to its effect on mTOR signaling (**Supplementary Figure 10**). These results thus suggest that AMPK activation is probably not the main mechanism of suppressive effects of high metformin concentration on T cells in contrast to the beneficial effects of low concentration of metformin shown to act via AMPK (24). The reduction in mTOR signaling is more likely to play a role, since metformin suppressed the S6RP phosphorylation in T cells from PBMC. However, other mechanisms directly linked to the metabolic effects of metformin likely play a role since the effects of metformin could not be fully recapitulated by rapamycin.

One of the key parameters that govern the success of an anti-tumor T cell response are the tumor microenvironment and the characteristics of T cells that enable their function in such an environment. The beneficial effect of metformin on anti-tumor immunity was at least partly attributed to its effects on tumor cells by reducing the hypoxia in the tumor microenvironment (13) and reducing the expression of PD-L1 on tumor cells (17). Metformin can also directly improve the T cell function in hypoxia (14) and increase T cell mitochondrial mass in some contexts (34, 54, 55) which could improve T cell metabolic fitness and therefore function in low glucose environments (51). Additionally, metformin can blunt the increased PD-1 expression on T cells (14). In our study, metformin treatment failed to increase the mitochondrial mass or maximal respiratory capacity of either Jurkat cells or T cells from PBMC, but 5 mM metformin did reduce the PD-1 expression in activated Jurkat cells. However, this was mostly the result of the reduced activation of Jurkat cells, as evidenced by the lack of a substantial decrease in the PD-1/CD69 expression ratio. However, even though metformin alone did not significantly reduce the PD-1 expression in T cells from PBMC, it did significantly reduce the PD-1/CD69 ratio in both CD4+ and CD8+ T cells (**Figure 9**). Interestingly, this effect was only observable with 5 mM and not 0.3 mM metformin. This observed change in the PD-1/CD69 ratio could indicate a shift in the balance of activation and PD-1 expression towards the former, which could be beneficial in the context of tumor immunology, particularly in conjunction with reduced PD-L1 expression on tumor cells (17). An ever expanding array of clinical and *in vivo* studies demonstrating the increased effectiveness of immune checkpoint inhibitor therapy in combination with metformin support this notion (13, 18–21, 67). The precise mechanism is still under investigation, and some reports highlight the improvement of the functionality of PD-1+ cells with metformin as a key factor (23).

### 4.2 The effect of glucose availability and insulin level on T cell function

Overall, the high (25 mM) glucose concentration itself did not substantially impact the survival or function of either Jurkat cells or T cells from PBMC, apart from a weak trend towards higher IFN-γ secretion in Jurkat cells. Interestingly, in Jurkat cells treated with 0.3 mM metformin, we observed a trend of increased IFN-γ secretion (not significant) as well as increased PD-1 expression in 25 mM versus 5.6 mM glucose, while the opposite effect was observed for IFN-γ with 5 mM metformin treatment. These observations are mostly consistent with the notion that high glucose levels can potentially lead to a more pro-inflammatory as well as exhausted and/or dysfunctional phenotype (22, 23, 65, 84), particularly in the presence of *in vivo* achievable metformin concentrations. However, the fact that most of the investigated parameters remained unchanged in high glucose conditions suggests that a prolonged exposure [as during diabetes (65)] is required for the effect to be fully expressed. Additionally, the effect of metformin itself remained mostly unchanged by and independent of the high glucose levels in both T cell models. Furthermore, despite the emerging role of insulin signaling in T cell function (85), the insulin level did not appear to alter the effect of metformin either. This suggests that the direct effect of metformin on T cells is not blunted by the high levels of glucose or insulin and so could be present even in patients where high blood glucose is not fully ameliorated. This is consistent with a recent study on a mice model where glucose levels were not normalized but metformin was nevertheless able to protect the obese mice from influenza-related mortality (22).

In contrast to high glucose, low glucose conditions substantially reduced the Jurkat cell survival, proliferation and IFN-γ secretion. The total cell number was reduced to about 50% with a significantly increased fraction of dead cells. No major difference in total cell number between 0.56 mM and 0 mM glucose indicates that glucose was completely consumed during the experiment in both cases. There was also a trend towards reduced activation and significant reduction in IFN-γ secretion in low glucose conditions in Jurkat cells. This is consistent with the reduced ATP production restricting activation, and the established role of glycolysis in IFN-γ secretion (45). T cells from PBMC were comparably less susceptible to low glucose levels, with a decreased percentage of proliferated cells (from 45% to 30%) and no significant effect on differentiation or cytokine secretion. On the contrary, CD69 expression was increased in low glucose. This is likely a reflection of considerable metabolic plasticity and spare capacity in key energy metabolism pathways in T cells from PBMC of healthy donors. While glycolysis plays a large role in T cell effector functions (45), T cell activation can be supported by oxidative metabolism (42), and healthy T cells can effectively reprogram their metabolism to function in low glucose environments (52, 86). We observed no difference in ATP production from oxidative phosphorylation and only a modest trend towards lower glycolytic ATP production in low glucose conditions. While the total ATP production in low glucose conditions also trended lower, it was apparently sufficient to support T cell activation and cytokine secretion, even though proliferation was reduced. Indeed, the increased CD69 expression and decreased PD-1/CD69 ratio in 0.56 mM versus 5.6 mM glucose means that despite the slower proliferation, healthy T cells might actually be less susceptible to inhibition via the PD-L1/PD-1 axis in low glucose environments.

In contrast, Jurkat cells of lymphoblastic malignant origin are very reliant on glycolysis with their total ATP production being reduced by about 70% in the absence of glucose. They also have very limited spare respiratory capacity, as demonstrated by their inability to substantially increase OxPhos ATP production in low glucose media or in response to FCCP injection (**Figure 2**; **Supplementary Figure 2**). This inability might be in part caused by their altered signaling pathways, particularly the deletion of the phosphatase and tensin homolog phosphatase that leads to constitutive activation of PI3K and Akt pathway (87). The constitutive activation of anabolic metabolism could thus make them unable to shift their metabolism to catabolism to increase ATP generation and lower metabolic demands.

### 4.3 2-deoxy-D-glucose improves effector functions in Jurkat cells and decreases PD-1 expression in T lymphocytes

In addition to ATP production, glycolysis is also a source of several important intermediates, including the substrates for protein N-glycosylation. This is relevant for the functions of immune receptors (56). N-glycosylation plays a particularly important part in stability and function of both PD-1 (62–64) and its ligand PD-L1 (59), making it a promising target for adjuvants in cancer immunotherapy (88). Several studies have shown that inhibition of PD-L1 glycosylation by metformin (via AMPK) (17), 2DG (57, 58) or their combination (60) can reduce PD-L1 expression on tumor cells. PD-1 surface expression on T cells can also be reduced by inhibition of protein N-glycosylation by 2-fluoro-L-fucose (89), tunicamycin or 2DG (60, 62). We therefore explored the effects of 2DG on the effector functions and PD-1 expression.

Interestingly, 2DG at physiologically achievable 0.6 mM concentration reduced surface PD-1 expression and substantially boosted IFN-γ secretion while IL-2 was mostly not affected. This effect was stronger than that on activation, as also evidenced by the reduced PD-1/CD69 ratio (**Figure 5**). Additionally, 2DG was able to suppress PD-L1 expression in Jurkat cells upon their activation (60). Since 0.6 mM 2DG only has a modest effect on glycolytic ATP production (60), its effects primarily result from its inhibition of protein N-glycosylation rather than energy stress. This is in contrast to low glucose where the effects were primary due to inhibition of glycolysis. This is also supported by the recapitulation of the effects of 2DG on the PD-1/PD-L1 axis by tunicamycin (60, 62). Higher, 4.8 mM concentration of 2DG that substantially inhibited glycolytic ATP production (60) also considerably decreased CD69 expression and IL-2 secretion following activation in Jurkat cells. In T cells from PBMC, it had an even stronger suppressive effect on CD69 expression and all subsequent effector functions, including proliferation as well as both IL-2 and IFN-γ secretion, than low glucose. Overall, our results show that the effects of low, physiologically achievable (0.6 mM) 2DG concentration on PD-1/PD-L1 axis and IFN-γ secretion could potentially improve T cell exhaustion without the excessive suppression of T cell effector functions (60).

### 4.4 The modulation of the effect of metformin on T cells by glucose availability

Treatment of Jurkat cells with 5 mM metformin in low glucose media led to a stronger reduction in total cell number, activation, IL-2 secretion and PD-1 expression. Notably, there was a significant and synergistic increase in cell death with 5 mM metformin versus control at 0 mM glucose (**Figure 1**), most likely reflecting a severe energy crisis caused by the blockade of both major ATP generating processes. Metformin and low glucose also synergistically reduced CD69 expression. However, in all other parameters, the effects of metformin and low glucose appeared essentially independent of each other and no synergism could be confirmed statistically. Thus, the effect of metformin itself in Jurkat cells appeared mostly independent of glucose levels. This is interesting in light of studies demonstrating the glucose-dependent effect of metformin in cancer cells. The most likely explanation for this discrepancy is the difference in spare ATP production capacity. While some other cancer cell types, for example MDA-MB-231 breast cancer cells, can compensate suppressed glycolysis or respiration with the other process to maintain sufficient ATP production (35, 60, 90), Jurkat cells lack substantial spare capacity in either and are thus already susceptible to a disruption of one of these processes.

On the other hand, glucose availability did importantly modify the effect of metformin on T cells from PBMC. While differentiation subsets were not affected in either CD4+ or CD8+ T cells, metformin strongly suppressed both the activation and PD-1 expression at 0.56 mM glucose, which was not the case at 5.6 mM or 25 mM glucose. Even though the PD-1/CD69 ratio was still substantially reduced, there was also a more pronounced reduction of IFN-γ secretion and a complete suppression of T cell proliferation by metformin at 0.56 mM glucose. In all of these cases (except for proliferation), there was significant statistical interaction between metformin and glucose. This is consistent with a recent report observing a reduction of IFN-γ secretion using low metformin concentration in combination with glycolysis inhibition by 2DG (25). Mechanistically, reducing the glycolytic flux with low glucose and suppressing the TCA cycle flux by blocking its main electron sink (the electron transfer chain) should result in reduced phosphoenolpyruvate levels (86), leading to a strong suppression of IFN-γ secretion as observed in our study. Metformin treatment in low glucose conditions also led to stronger average reduction in Eomes and T-bet expression, even though no synergism could be confirmed statistically. Still, these results are consistent with the stronger suppression of IFN-γ in low glucose.

Additionally, reducing glycolysis and blocking respiration with metformin strongly reduced the ability of T cells to generate ATP (**Figure 8**), further restricting the amount of ATP available for the metabolically demanding processes of activation, proliferation and cytokine secretion. Such restricted energy generating capacity should also lead to energy stress and AMPK activation known to be able to reduce IFN-γ secretion (52). While AMPK is likely to be strongly activated by metformin in low glucose conditions, this is unlikely to explain its suppressive effects other than reduced CD69 expression in Jurkat cells due to the effects of AMPK inhibitor compound C discussed above and our result using AMPK activator A 769662 (data not shown). mTOR signaling was also further reduced by metformin treatment in low glucose conditions (**Figure 11**), helping to explain the observed reduction in proliferation. Furthermore, reducing both glycolysis and mitochondrial metabolism is likely to directly limit the amount of ATP and biosynthetic intermediates available for cell proliferation (36) and cytokine secretion. This is also supported by the observation of stronger suppressive effects of combined metformin and 2DG treatment on primary T cell activation and function compared to either drug alone (53). Overall, our results indicate that the suppressive effect of high metformin concentration on T cells is much stronger in low glucose conditions. Some caution is therefore warranted when using high concentrations of metformin on T cells in low glucose contexts, and further studies are needed on the subject.

Comparing the two T cell models used in the study, there was a degree of agreement between the results on Jurkat cells and T cells from PBMC of healthy donors, e.g. in the effect of metformin on proliferation and the overall lack of effect of high glucose conditions. On the other hand, Jurkat cells were more susceptible to metformin treatment and particularly low glucose levels, with the notable exceptions of IFN-γ secretion and the PD-1/CD69 ratio. It should also be noted that the effects of glucose availability in particular was dependent on the method of activation used to activate Jurkat cells, with the effects of low glucose on CD69 and PD-1 expression as well as on IL-2 secretion were much more pronounced when PMA/ionomycin was used instead of anti-CD3/anti-CD28 antibodies (**Supplementary Figure 3**). Altogether, Jurkat cells present a simple model of T cells that enable fast testing of various metabolic drugs, however other more complex models such as PBMC and *in vivo* models are necessary to understand the mechanisms of actions of metformin and other metabolic inhibitors.

## 5 Conclusions

**Figure 12:**
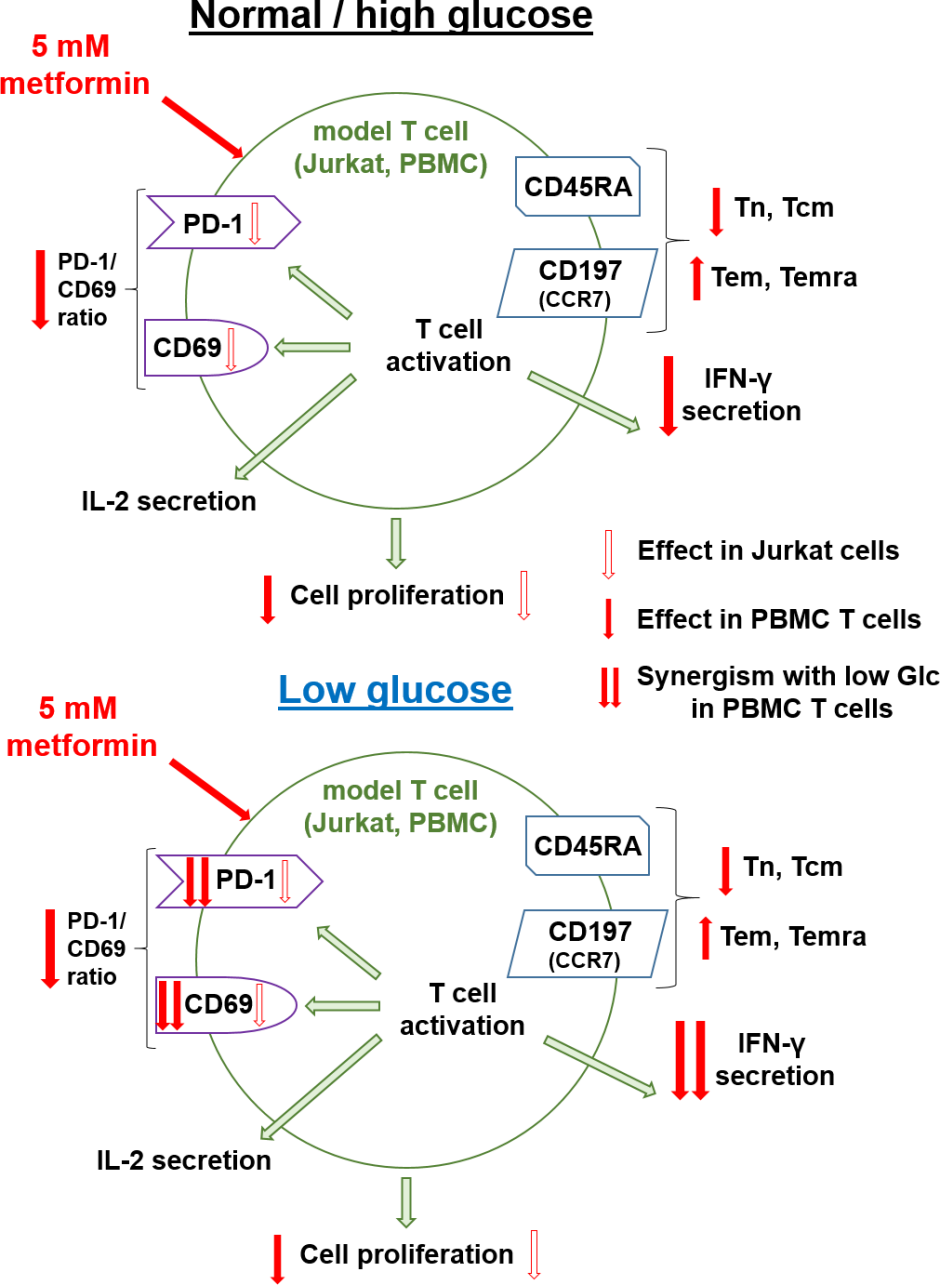
Summary of the effects of metformin on Jurkat cells and activated T cells from PBMC in normal/high and low glucose. Red arrows with white filling indicate the effect of metformin in Jurkat cell while full red arrows indicate the effect of metformin in T cells from PBMC. Double red arrows indicate synergistic effects of metformin and low glucose in T cells from PBMC.

In the present study, we investigated the effects of metformin on Jurkat cells and T cells from PBMC as a function of glucose availability. At the normal physiological level of glucose, low (0.3 mM) concentration of metformin had a very limited suppressive effect on Jurkat cell function and did not affect activation and cytokine secretion in T cells from PBMC, consistent with previous studies. On the other hand, higher (5 mM) concentration of metformin used to mimic *in vivo* accumulation with chronic use, reduced T cell proliferation in both models, as well as activation in Jurkat cells and IFN-γ secretion in T cells from PBMC (**Figure 12**). These effects were associated with reduced ATP production from oxidative phosphorylation compared to control that was fully compensated by glycolysis only in resting (unactivated) PBMC, but not in activated primary T cells or Jurkat cells. Additionally, high but not low metformin concentration reduced mTOR signaling and reduced the expression of transcription factors T-bet, Eomes and STAT3 that likely played a role in reduced proliferation and cytokine secretion. Despite these suppressive effects, high but not low metformin reduced PD-1 expression in Jurkat cells and improved the PD-1/CD69 expression ratio in primary T cells, potentially improving the balance between T cell activation and exhaustion. T cell differentiation was also shifted from naïve and memory to effector subsets, suggesting potentially improved capacity for effector functions at the cost of T cell persistence and proliferation potential. Overall, our results demonstrate that high concentrations of metformin often used in cancer metabolism research *in vitro* can also have a suppressive effect on T cells, and so warrant additional study.

Glucose level had a variable effect on model T cells. High, hyperglycemic glucose concentration had a very limited effect in both T cell models and did not affect the response of T cells to metformin, indicating that the direct effects of metformin on T cell function should still be present in patients with poor glucose control. On the other hand, low glucose level reduced Jurkat cell proliferation without improving the exhaustion/activation balance. Primary T cells from PBMC were a lot more resistant to the effects of low glucose and maintained CD69 expression as well as cytokine secretion, with only proliferation being adversely affected. Interestingly, low glucose even increased CD69 expression and improved the PD-1/CD69 expression ratio, suggesting T cells from PBMC might be less susceptible to inhibition via PD-1/PD-L1 axis in low glucose conditions. The suppressive effects on T cell function were stronger with metformin treatment in low glucose conditions in both models. In Jurkat cells, this appeared to be mostly the result of two additive suppressive effects on cellular energetics. In T cells from PBMC, low glucose and metformin treatment had a strong synergistic effect on CD69 and PD-1 expression as well as on IFN-γ secretion. While the effects of metformin and low glucose on proliferation were not synergistic, they together almost completely suppressed T cell proliferation. However, the shift to effector T cell subsets with metformin treatment was completely independent of glucose levels. Overall, most of the suppressive effects of metformin can be potentiated in low glucose conditions such as in the tumor microenvironment.

Finally, we show that 2DG, especially at low, physiologically achievable concentrations that do not substantially inhibit glycolysis, was able to reduce the PD-1/CD69 ratio and stimulate IFN-γ secretion while mostly preserving IL-2 secretion in Jurkat cells, suggesting potential utility in boosting anti-cancer immunity.

## Data availability statement

The raw data supporting the conclusions of this article will be made available by the authors, without undue reservation.

## Ethics statement

The studies involving humans were approved by Medical Ethic Committee of the Republic of Slovenia (Komisija Republike Slovenije za medicinsko etiko), Štefanova ulica 5, 1000 Ljubljana, Slovenia. The studies were conducted in accordance with the local legislation and institutional requirements. The participants provided their written informed consent to participate in this study.

## Author contributions

MP and HS contributed to the conception of the study. MP designed and supervised the study. MP and JR developed the methodology. JR performed the experiments and acquired the data on Jurkat cells. JR and LP performed the experiments and acquired the data on PBMC. JR performed the statistical analysis and data visualisation. JR and MP wrote the manuscript. All authors contributed to the article and approved the submitted version.
