# Supplementary material for: Low glucose availability potentiates the effects of metformin on model T cell activation and exhaustion markers in vitro
Source: Front Endocrinol (Lausanne). 2023 Dec 5;14:1216193. doi: 10.3389/fendo.2023.1216193 (PMC10728603; doi:10.3389/fendo.2023.1216193)
Supplement: Supplementary file 1 [file DataSheet_1.pdf]

frontiers

Supplementary Material

The interaction between glucose availability and the effects of metformin on model T lymphocyte exhaustion and activation markers

Jernej Repas[1], Lea Peternel[1], Harald Sourij[2*], Mojca Pavlin[1,3*]

[1]Institute of Biophysics, Faculty of Medicine, University of Ljubljana, Ljubljana, Slovenia

[2]Trials Unit for Interdisciplinary Metabolic Medicine, Division of Endocrinology and Diabetology, Medical University Graz, Graz, Austria

[3]Group for nano- and biotechnological applications, Faculty of electrical engineering, University of Ljubljana, Ljubljana, Slovenia

*** Correspondence:**
Assist. Prof. Mojca Pavlin, PhD
mojca.pavlin@mf.uni-lj.si

Prof. Harald Sourij

ha.sourij@medunigraz.at

Keywords: T cells, metformin, glucose level, 2-deoxy-D-glucose, T cell exhaustion, PD-1/PD-L1 axis

Abstract

Modulation of immune cell metabolism is one of promising strategies to improve cancer immunotherapies. Metformin is an anti-diabetic drug with potential anti-cancer effects, ranging from normalization of blood glucose and insulin levels, direct anti-proliferative effects on cancer cells to emerging immunomodulatory effects on anti-tumor immunity. Metformin can reduce tumor hypoxia and PD-L1 expression, as well as normalize or improve of T cell function and potentiate the effect of immune checkpoint inhibitors, making it a promising adjuvant to immunotherapy of tumors with poor response such as triple negative breast cancer (TNBC). However, although the effects of metformin on cancer cells are glucose-dependent, the role of glucose in modulating its effect on T cells has not been systematically studied. We thus investigated the effect of metformin as a function of glucose level on Jurkat cell and PBMC T cell models in vitro. While low metformin concentrations had little effect on T cell function, high concentration reduced proliferation and IFN-γ secretion in both models and induced a shift in T cell populations from memory to effector subsets. The PD-1/CD69 ratio was improved by high metformin in T cells from PBMCs. Low glucose and metformin synergistically reduced PD-1 and CD69 expression and IFN-γ secretion in T cells from PBMCs. Low glucose level itself suppressed Jurkat cell function due to their limited metabolic plasticity, but had limited effects on T cells from PBMCs apart from reduced proliferation. Conversely, high glucose did not strongly affect either T cell model. Metformin in combination with glycolysis inhibitor 2-deoxy-D-glucose reduced PD-1 in Jurkat cells, but also strongly suppressed their function. However, low, physiologically achievable 2DG concentration itself reduced PD-1 while mostly maintaining IL-2 secretion and, interestingly, even strongly increased IFN-γ secretion regardless of glucose level. Overall, glucose metabolism can importantly influence some of the 2 This is a provisional file, not the final typeset article effects of metformin on T cell functionality in tumor microenvironment. Additionally, we show that 2DG could potentially improve the anti-tumor T cell response.

1 Supplementary Figures and Tables

1.1 Supplementary Figures

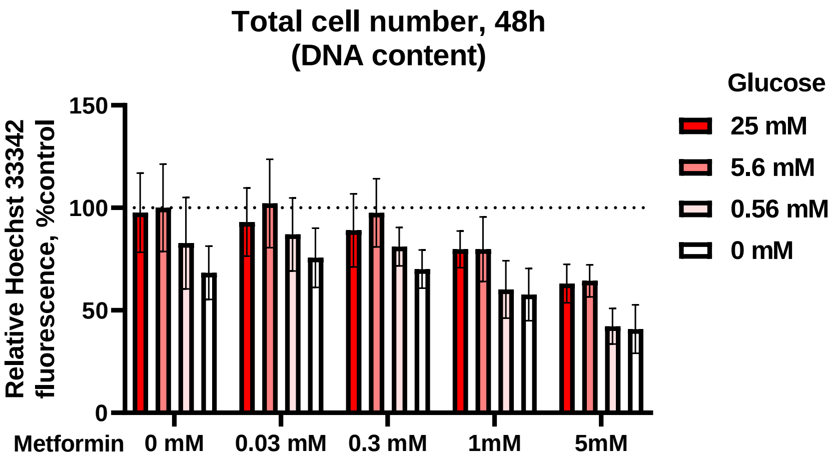

Supplementary Figure 1: The effect of metformin treatment and glucose availability on Jurkat cell survival and proliferation. Jurkat cells were grown in media with different glucose concentrations and treated with different metformin concentrations as indicated. The total number of cells was determined after 48 h by Hoechst 33342 staining. The mean ± SEM is shown for two independent experiments. Two-way ANOVA did not confirm any synergism between the effects of glucose and metformin.

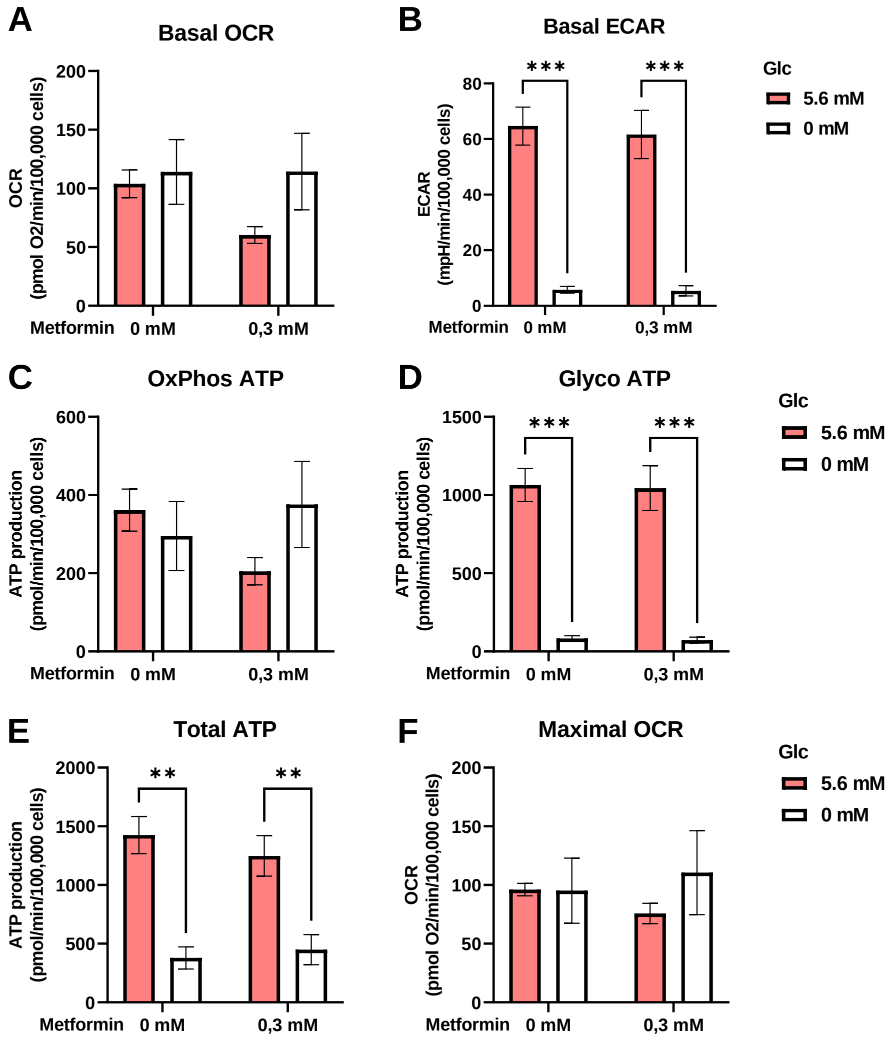

Supplementary Figure 2: The effect of metformin treatment on Jurkat cell energy metabolism in low vs. normal glucose conditions. Jurkat cells were grown in media supplemented with 0 mM or 5.6 mM glucose and treated for 48h with 0 mM or 0.3 mM metformin as indicated. Baseline OCR (**A**) and ECAR (**B**) as well as maximal OCR after FCCP injection (**F**) were determined using Seahorse Mito Stress Test assay. The ATP production from oxidative phosphorylation (**C**) and glycolysis (**D**), as well as total ATP production (**E**) were calculated according to the manufacturer's instructions for Seahorse Real Time ATP Assay. Mean ± SEM is shown for three independent experiments. $^{**}p<0.01$, $^{***}p < 0.001$ as determined by two-way ANOVA with Šidak's post-hoc test. No significant interaction between metformin and glucose level was found.

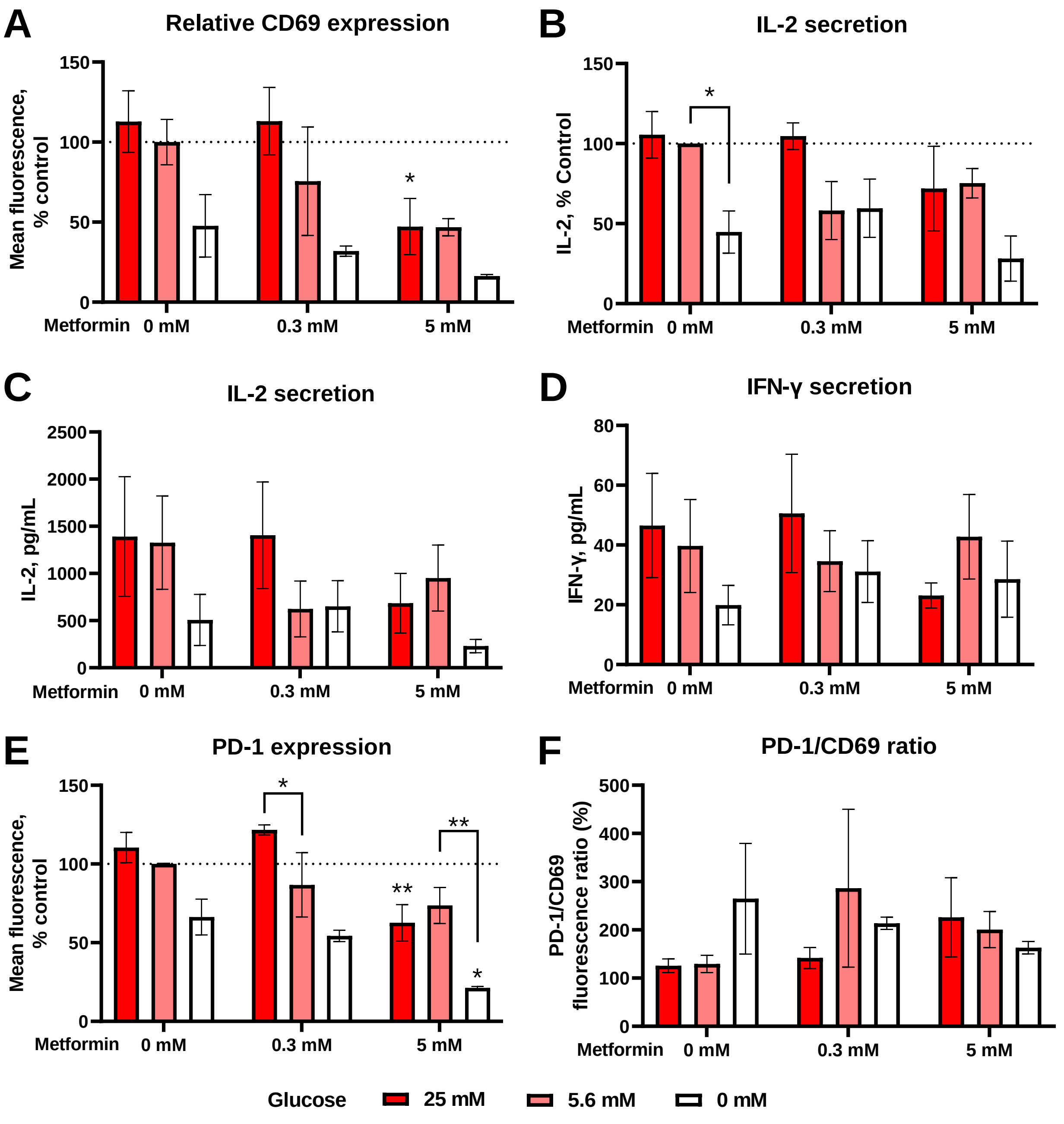

Supplementary Figure 3: The effect of metformin treatment and glucose availability on activation, cytokine secretion and PD-1 expression in Jurkat cells activated with PMA and ionomycin. Jurkat cells were activated with 25 ng/mL PMA and 1 μM ionomycin and grown for 24 h in media with different glucose (denoted by shades of red) and metformin concentrations. The expression of CD69 (**A**, **F**) and PD-1 (**E**, **F**) was determined by flow cytometry. The concentration of IL-2 (**B, C**) and IFN-γ (**D**) in the medium was determined by ELISA. The mean ± SEM is shown for three (**A**, **E, F**) or four (**B**, **C, D**) independent experiments. *p<0.05, **p<0.01 as determined by two-way ANOVA with Dunnett's or Šidak's post hoc test. Two-way ANOVA revealed no significant interaction between glucose level and metformin.

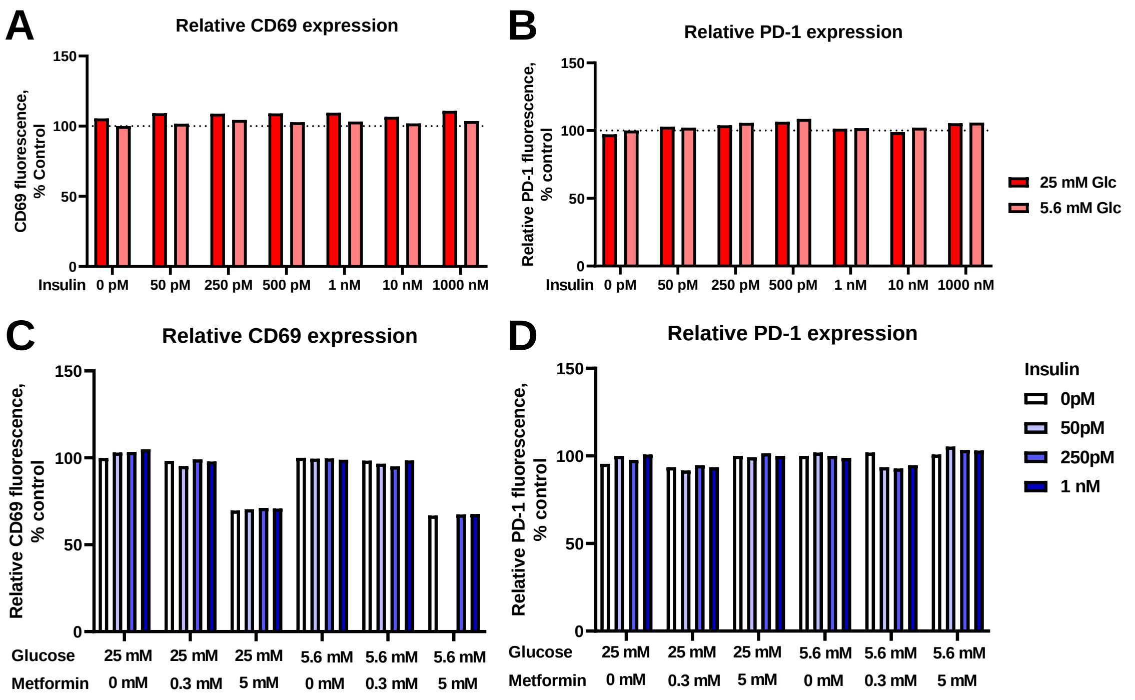

Supplementary Figure 4: The effect of insulin on CD69 and PD-1 expression in activated Jurkat cells. Jurkat cells were grown in media with different glucose (denoted by shades of red in **A-B**), insulin (shades of blue in **C-D**) and metformin (**C, D**) concentrations for 24 h. The cells were activated with PMA/ionomycin and relative surface expression of CD69 (**A, C**) and PD-1 (**B, D**) was determined by flow cytometry. The mean fluorescence is shown for one independent experiment.

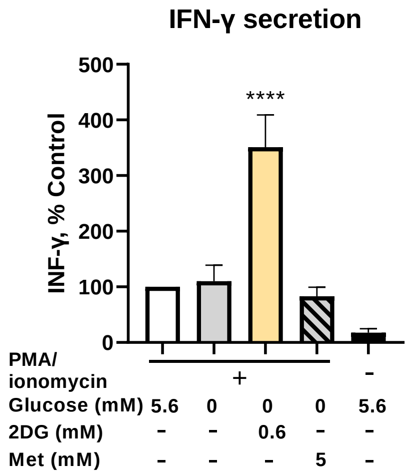

Supplementary Figure 5: The effect of metformin and 2-deoxy-D-glucose (2DG) treatment and glucose availability on IFN-γ secretion in activated Jurkat cells. Jurkat cells were activated with PMA/ionomycin and grown for 24 h in media with 0 mM or 5.6 mM glucose as indicated and treated with 5 mM metformin or 0.6 mM 2DG. The concentration of IFN-γ in the medium was determined by ELISA. The mean ± SEM is shown for four independent experiments. ****$p<0.0001$ vs. control as determined by ANOVA with Dunnett's post-hoc test. Data adapted from (1) according to Creative Commons Attribution license (http://creativecommons.org/licenses/by/4.0/).

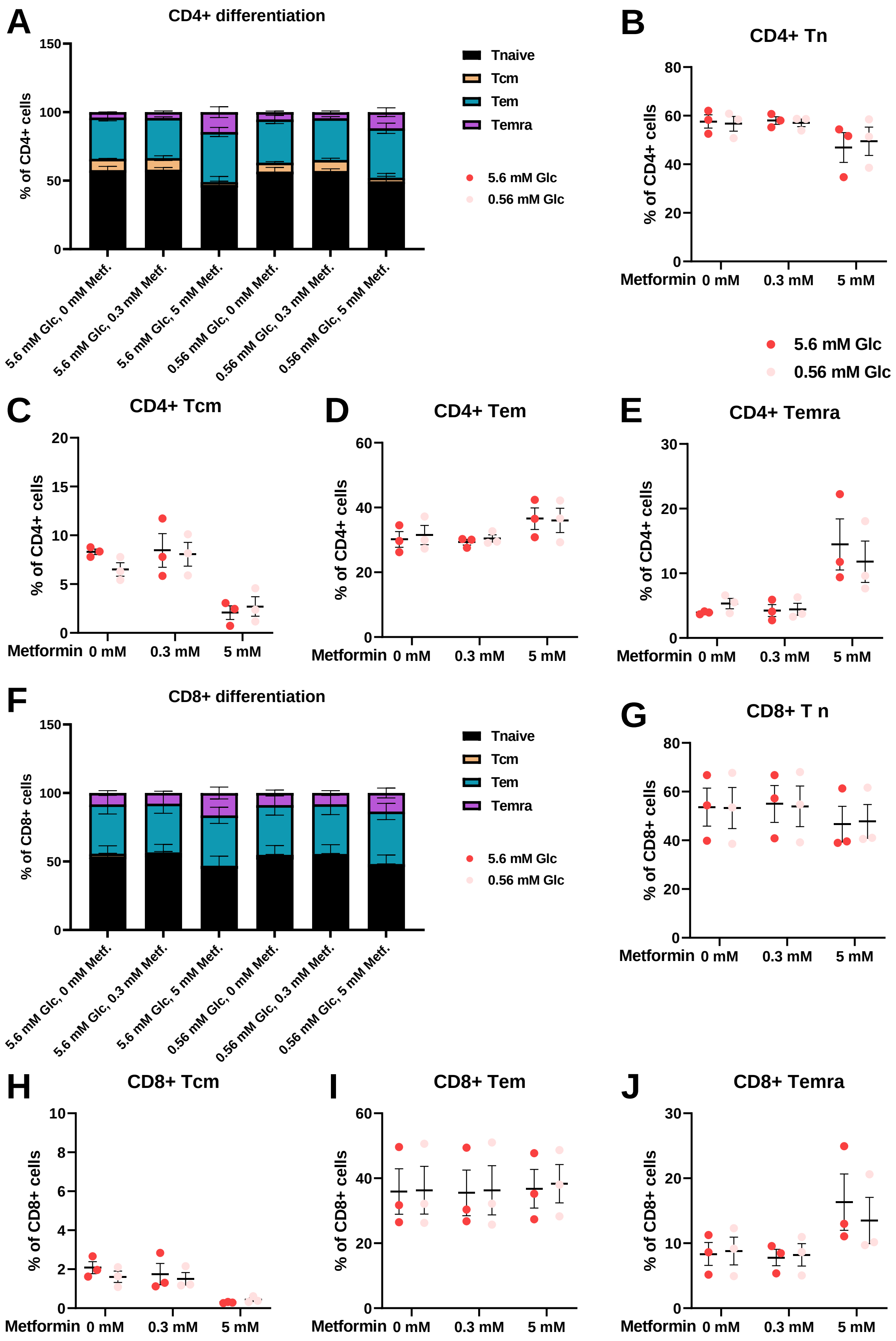
A
CD4+ differentiation
% of CD4+ cells
Tnaive
Tcm
Tem
Temra
5.6 mM Glc
0.56 mM Glc
5.6 mM Glc, 0 mM Metf.
5.6 mM Glc, 0.3 mM Metf.
5.6 mM Glc, 5 mM Metf.
0.56 mM Glc, 0 mM Metf.
0.56 mM Glc, 0.3 mM Metf.
0.56 mM Glc, 5 mM Metf.
B
CD4+ Tn
% of CD4+ cells
Metformin 0 mM 0.3 mM 5 mM
5.6 mM Glc
0.56 mM Glc
C
CD4+ Tcm
% of CD4+ cells
Metformin 0 mM 0.3 mM 5 mM
D
CD4+ Tem
% of CD4+ cells
Metformin 0 mM 0.3 mM 5 mM
E
CD4+ Temra
% of CD4+ cells
Metformin 0 mM 0.3 mM 5 mM
F
CD8+ differentiation
% of CD8+ cells
Tnaive
Tcm
Tem
Temra
5.6 mM Glc
0.56 mM Glc
5.6 mM Glc, 0 mM Metf.
5.6 mM Glc, 0.3 mM Metf.
5.6 mM Glc, 5 mM Metf.
0.56 mM Glc, 0 mM Metf.
0.56 mM Glc, 0.3 mM Metf.
0.56 mM Glc, 5 mM Metf.
G
CD8+ T n
% of CD8+ cells
Metformin 0 mM 0.3 mM 5 mM
H
CD8+ Tcm
% of CD8+ cells
Metformin 0 mM 0.3 mM 5 mM
I
CD8+ Tem
% of CD8+ cells
Metformin 0 mM 0.3 mM 5 mM
J
CD8+ Temra
% of CD8+ cells
Metformin 0 mM 0.3 mM 5 mM

Supplementary Figure 6 (previous page): The effect of low versus high metformin concentration as a function of glucose availability on CD4+ and CD8+ T cell differentiation in PBMC. PBMCs were treated for 72h with metformin in media supplemented with 5.6 mM or 0.56 mM glucose (denoted by shades of red). After treatment, the cells were stained with the appropriate antibodies and analyzed by flow cytometry. The CD3+ CD4+ (**A**-**E**) and CD3+ CD8+ (**F**-**J**) populations were divided into four populations according to CD45RA and CCR7 (CD197) expression: naïve T cells (Tnaive, CD45RA+ CCR7+, **B**, **G**), central memory T cells (Tcm, CD45RA- CCR7+, **C**, **H**), effector memory T cells (Tem, CD45RA- CCR7-, **D**, **I**) and terminally differentiated effector T cells (Temra, CD45RA+ CCR7-, **E**, **J**). The percentage of total CD3+ CD4+ or CD3+ CD8+ cells is shown for each subpopulation. The data points represent individual experiments, while the horizontal lines and error bars represent the mean percentage ± SEM. One independent experiment was performed for each of the three healthy donors. The distribution of subpopulations according to differentiation status is summarized in (**A**) and (**F**) by displaying mean percentages of the subsets for CD4+ and CD8+ T cells, respectively.

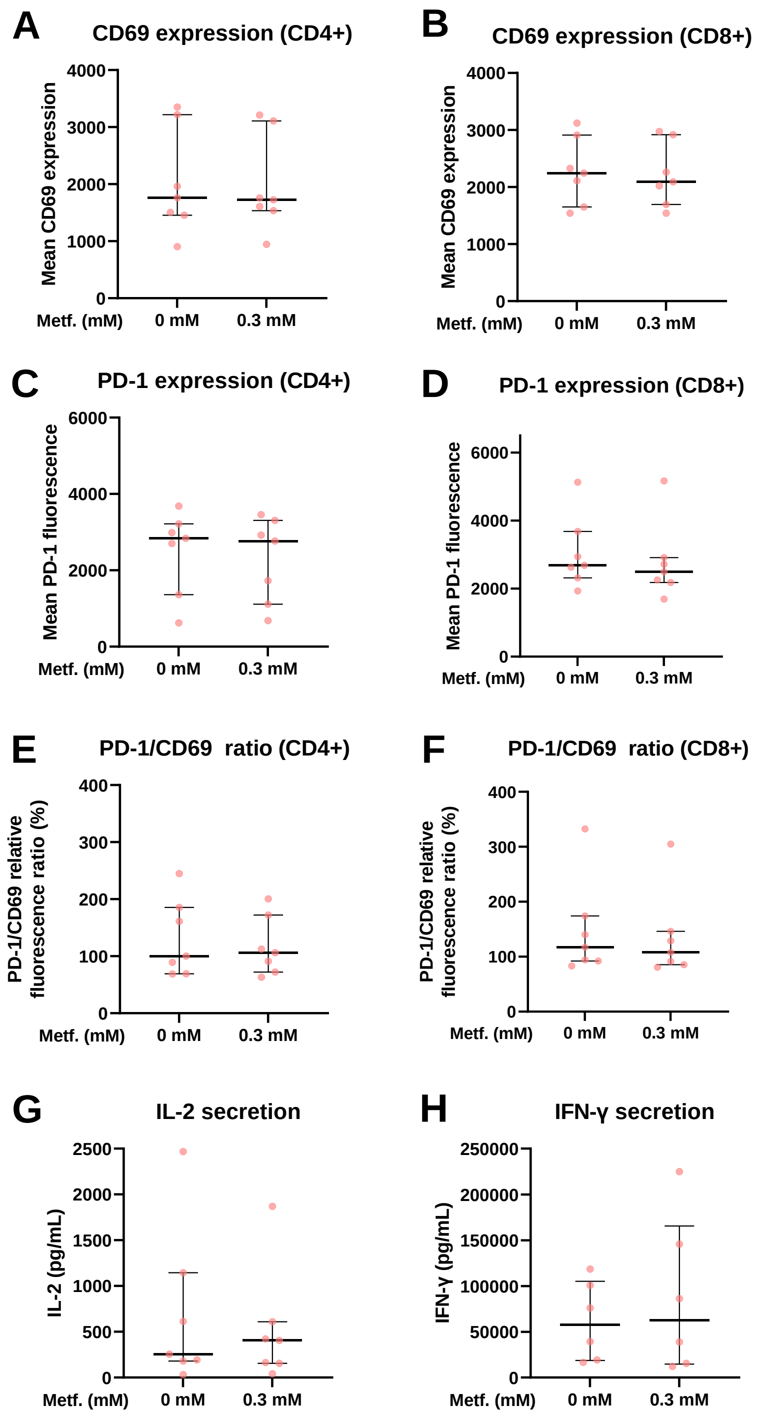

Supplementary Figure 7: The effect of low metformin concentration on T cell activation, PD-1 expression and cytokine secretion in activated T cells from PBMC. PBMCs were treated for 72h with 0.3 mM in media supplemented with 5.6 mM glucose and activated with anti-CD3 and anti-CD28 antibodies during the treatment. After treatment, the culture media were collected and the cells stained with antibodies for flow cytometry. The expression levels of activation marker CD69 (**A, B**), exhaustion marker/immune checkpoint PD-1 (**C, D**) and the ratio of PD-1/CD69 fluorescence (**E, F**) were determined in CD4+ (**A, C, E**) and CD8+ (**B, D, F**) T cells. The concentration of IL-2 after 24h treatment (**G**) and IFN-γ after 72h treatment (H) in culture media was determined with ELISA. The data points represent individual experiments, while the horizontal lines and error bars represent the median ± interquartile range. One independent experiment was performed for each of the six to seven healthy donors. Student's paired t-test revealed no significant differences.

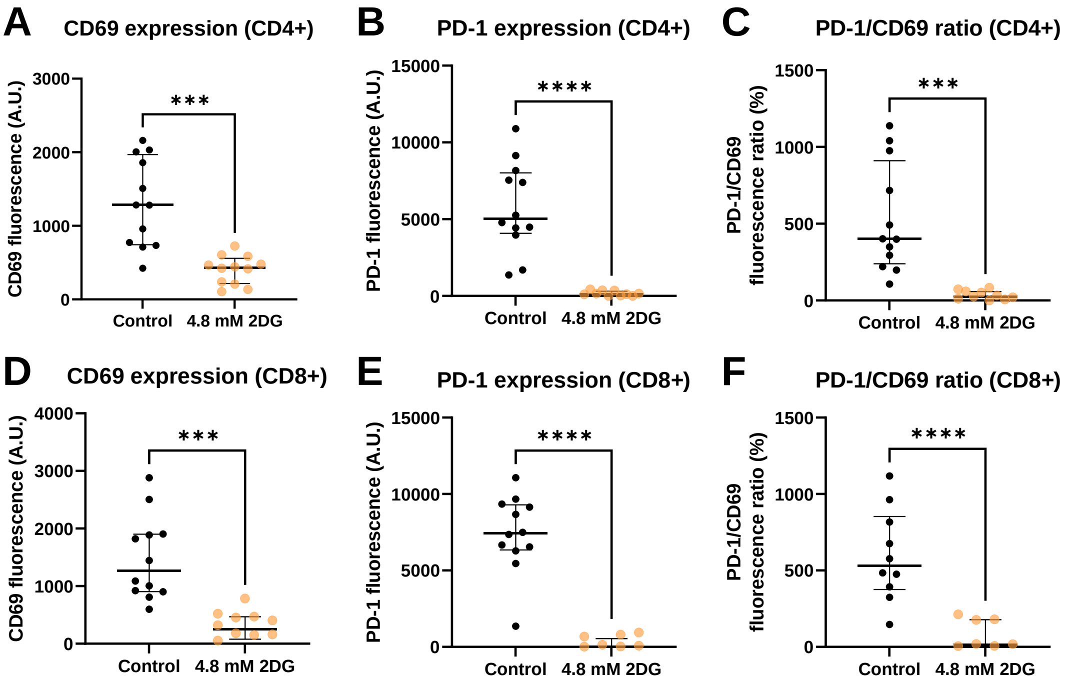

Supplementary Figure 8: The effect of 2-deoxy-D-glucose on T cell activation and PD-1 expression in activated T cells from PBMC. PBMCs were treated for 72h with 4.8 mM 2DG in medium supplemented with 5.6 mM glucose and activated with anti-CD3 and anti-CD28 antibodies during the treatment. After treatment, the cells were stained with antibodies and analyzed by flow cytometry. The expression levels of activation marker CD69 (**A, D**), exhaustion marker/immune checkpoint PD-1 (**B, E**) and the ratio of PD-1/CD69 fluorescence (**C, F**) were determined in CD4+ (**A-C**) and CD8+ (**D-F**) T cells. The data points represent individual experiments, while the horizontal lines and error bars represent the median ± interquartile range. Three independent experiments were performed for each of the four healthy donors for a total of twelve independent experiments. ***p<0.001, ****p<0.0001 as determined by Student's paired t-test.

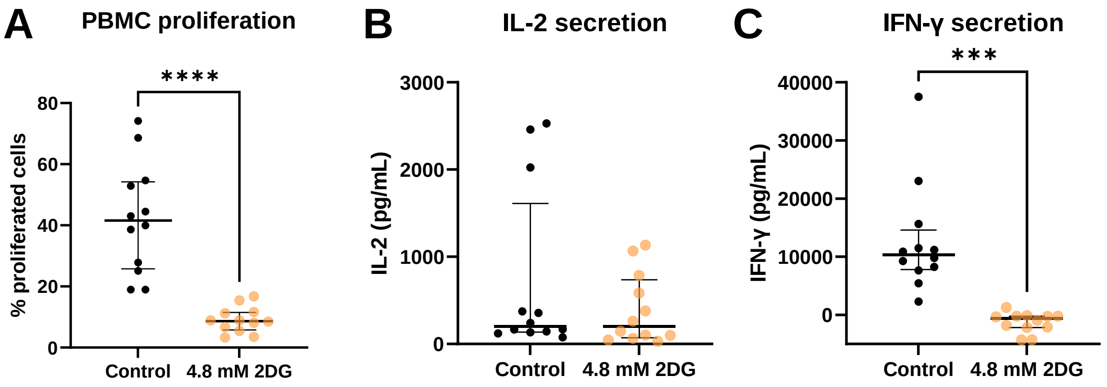

Supplementary Figure 9: The effect of 2-deoxy-D-glucose on proliferation and cytokine secretion in activated T cells from PBMC. PBMCs were treated for 72h with 2DG in medium supplemented with 5.6 mM glucose and activated with anti-CD3 and anti-CD28 antibodies during the treatment. (**A**) The percentage of proliferated PBMC cells following T cells activation was determined by measuring CFSE dilution. (**B, C**) The concentration of secreted IL-2 after 24h treatment (**B**) and IFN-γ in after 72h treatment (**C**) in culture media was determined with ELISA. The data points represent individual experiments, while the horizontal lines and error bars represent the median percentage ± interquartile range. Three independent experiments were performed for each of the four healthy donors for a total of twelve independent experiments. ***$p<0.001$, ****$p<0.0001$ as determined by Student's paired t-test.

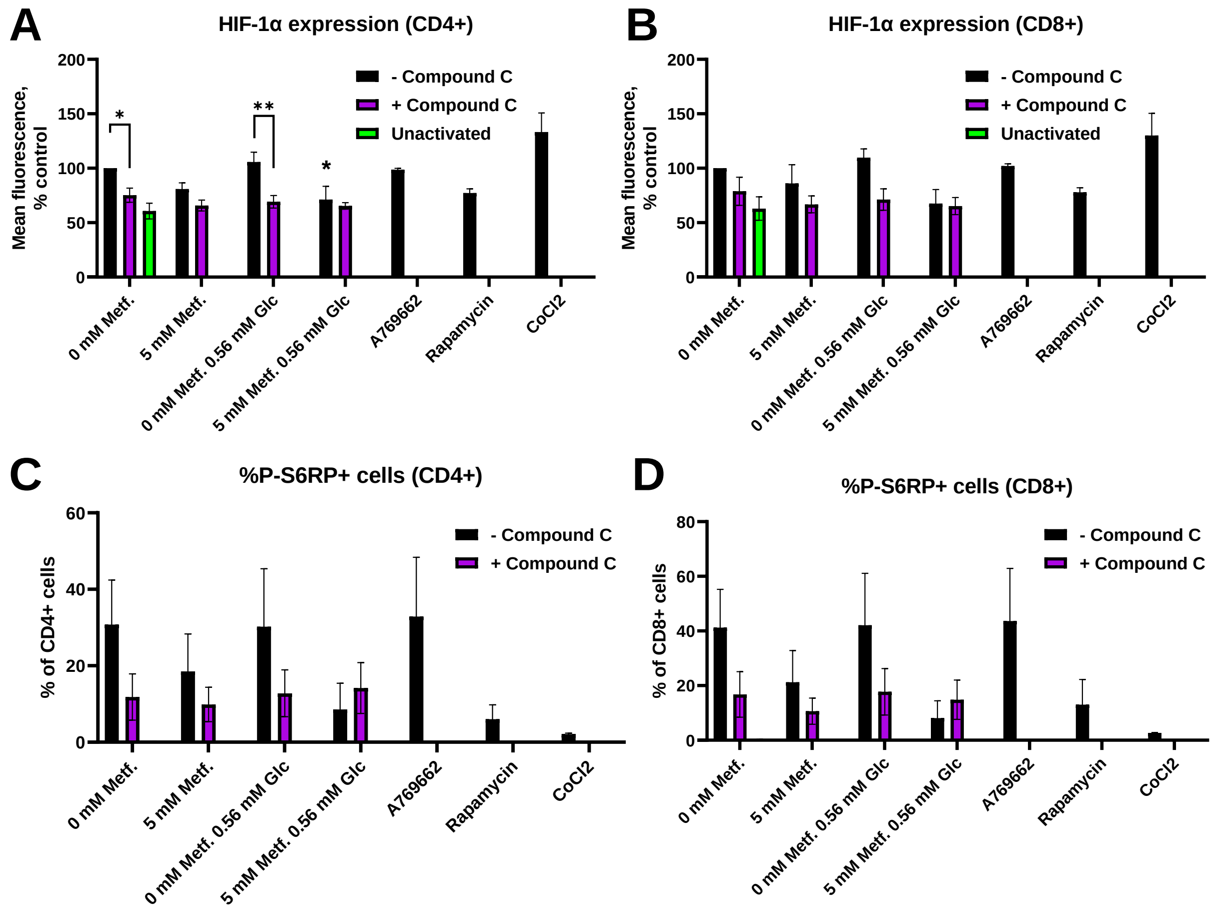

Supplementary Figure 10: The role of AMPK in the effect of metformin as a function of glucose availability on HIF-1α expression and mTOR signaling in activated T cells from PBMC. PBMCs were pretreated with 5 µM compound C for 30 min (denoted by purple bars) and subsequently treated for 48h with 5 mM metformin as indicated in media supplemented with 5.6 mM or 0.56 mM glucose and activated with anti-CD3 and anti-CD28 antibodies during the treatment. The expression of HIF-1α (**A**, **B**) and phosphorylated S6RP (**C**, **D**) was measured by intracellular staining flow cytometry. The mean ± SEM is shown for three independent experiments, one per donor. *p<0.05, **p<0.01 as determined by mixed effects model (**C**-**D**) or two-way ANOVA (**A, B**) with Dunnett'sss or Šidak's post-hoc test.

1.2 Flow cytometry gating strategies

1.2.1 Figure 1

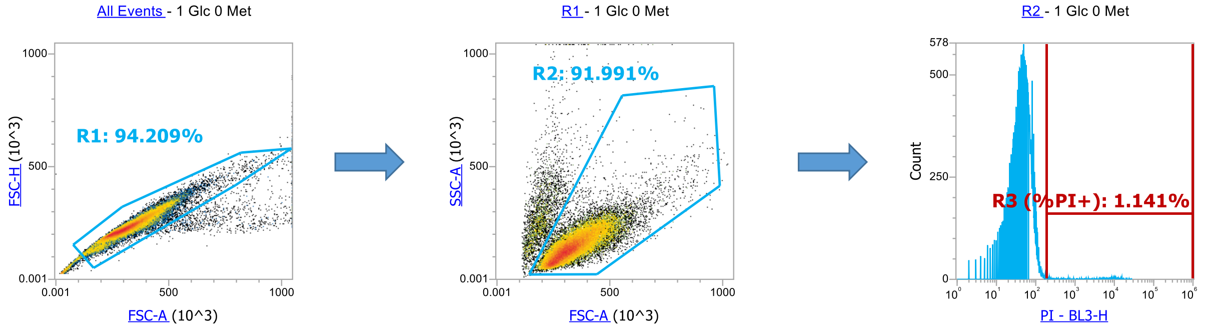

1.2.2 Figure 3

1.2.2.1 CD69 expression

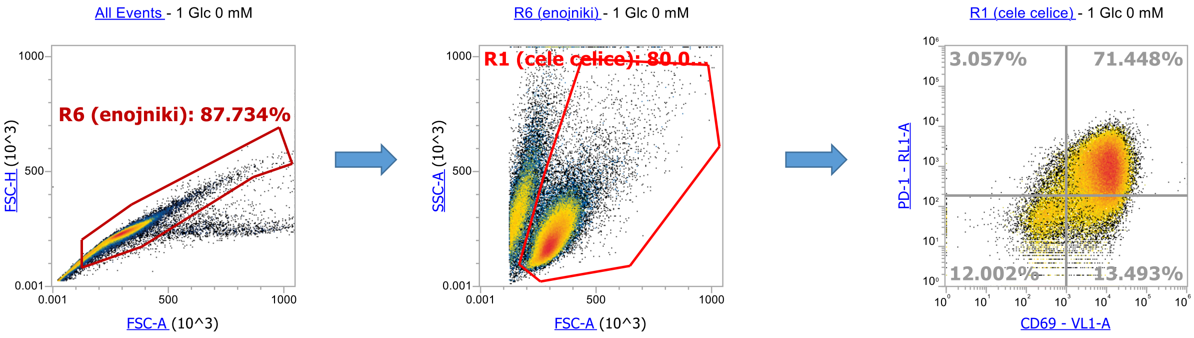

1.2.3 Figure 4

1.2.3.1 CD69 and PD-1 expression

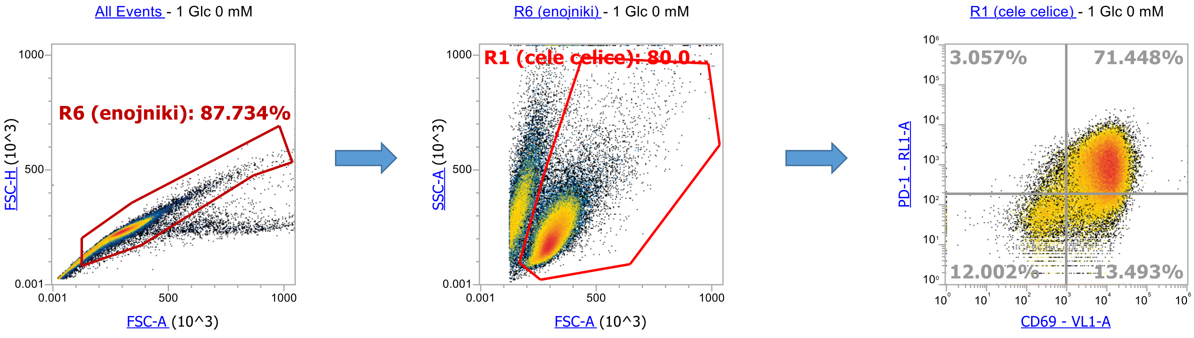

1.2.3.2 Mitochondrial mass (NAO)

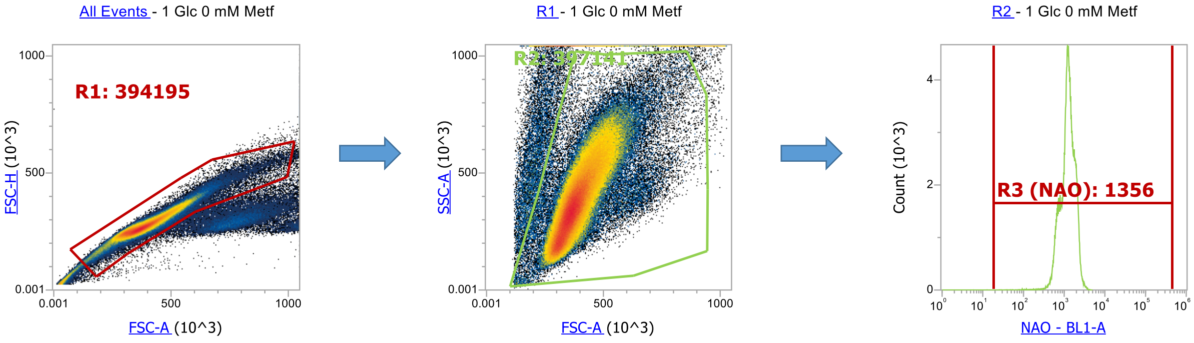

1.2.3.3 Mitochondrial mass (Mitotracker Orange)

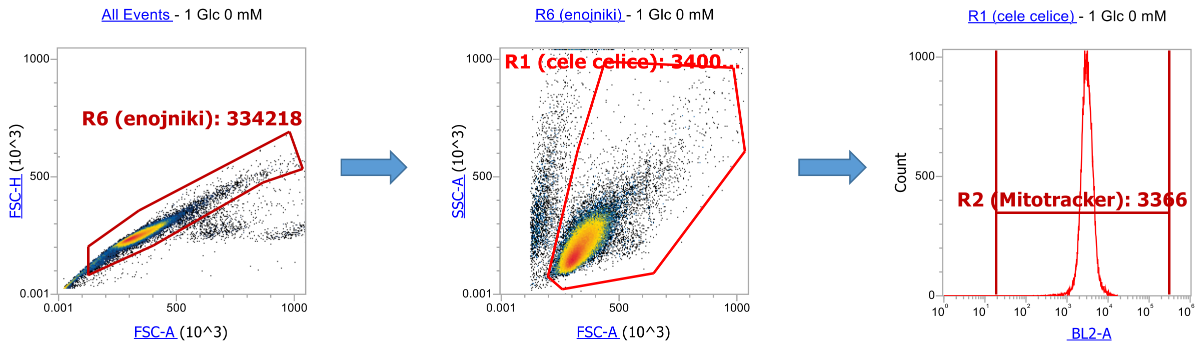

1.2.4 Figure 6 & 7

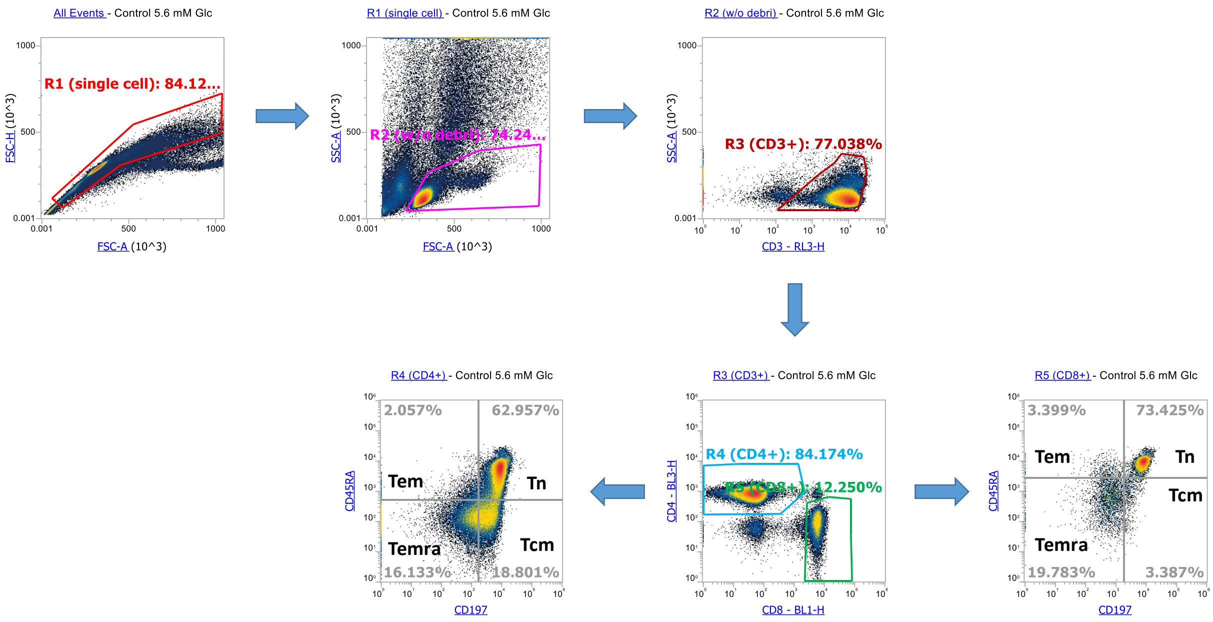

1.2.5 Figure 9

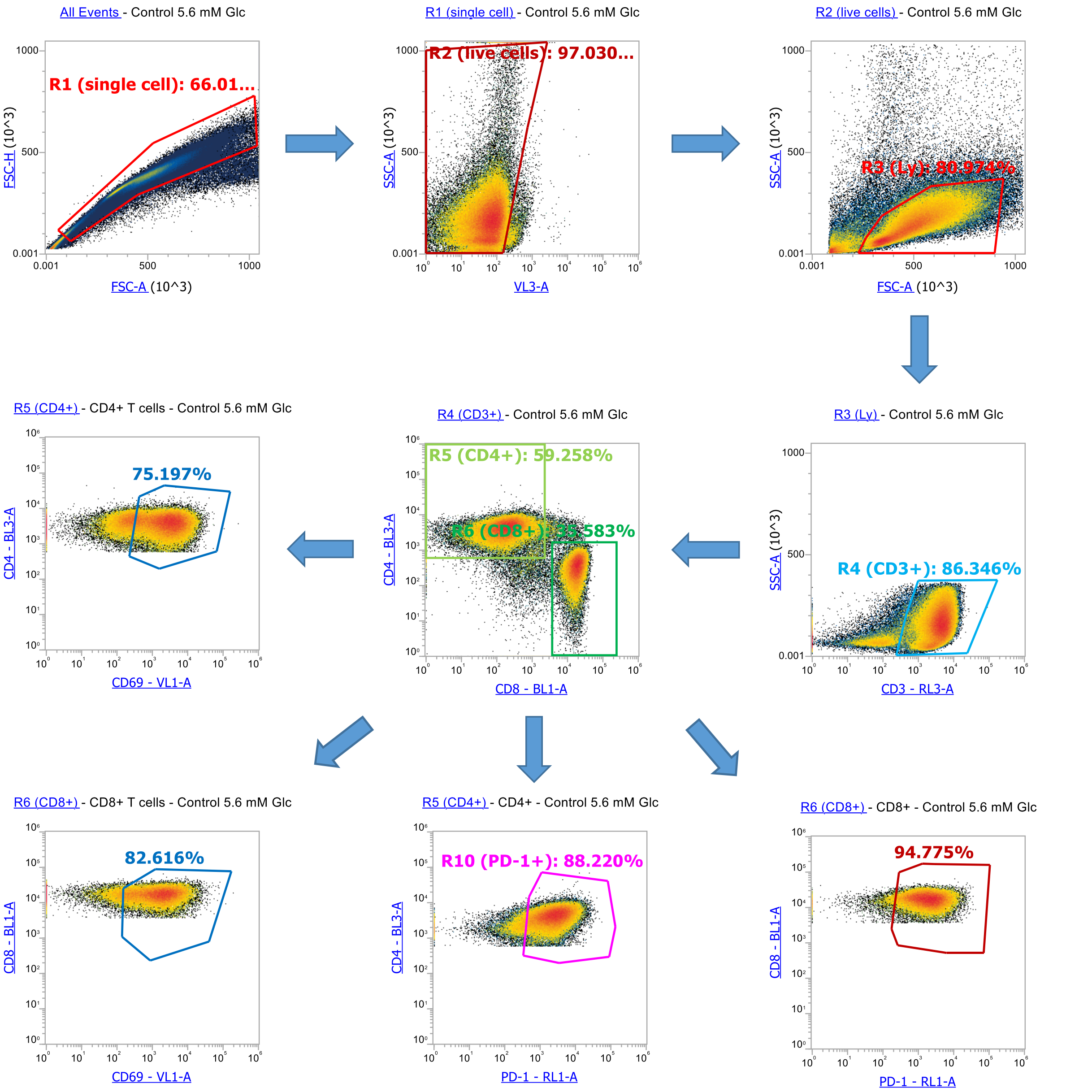

Zombie Yellow® was used as a viability die (seen in channel VL-3). The CD69+ and PD-1+ gates were set according to FMO controls.

1.2.6 Figure 10 &11

1.2.6.1 T-bet, Eomes & STAT3 expression

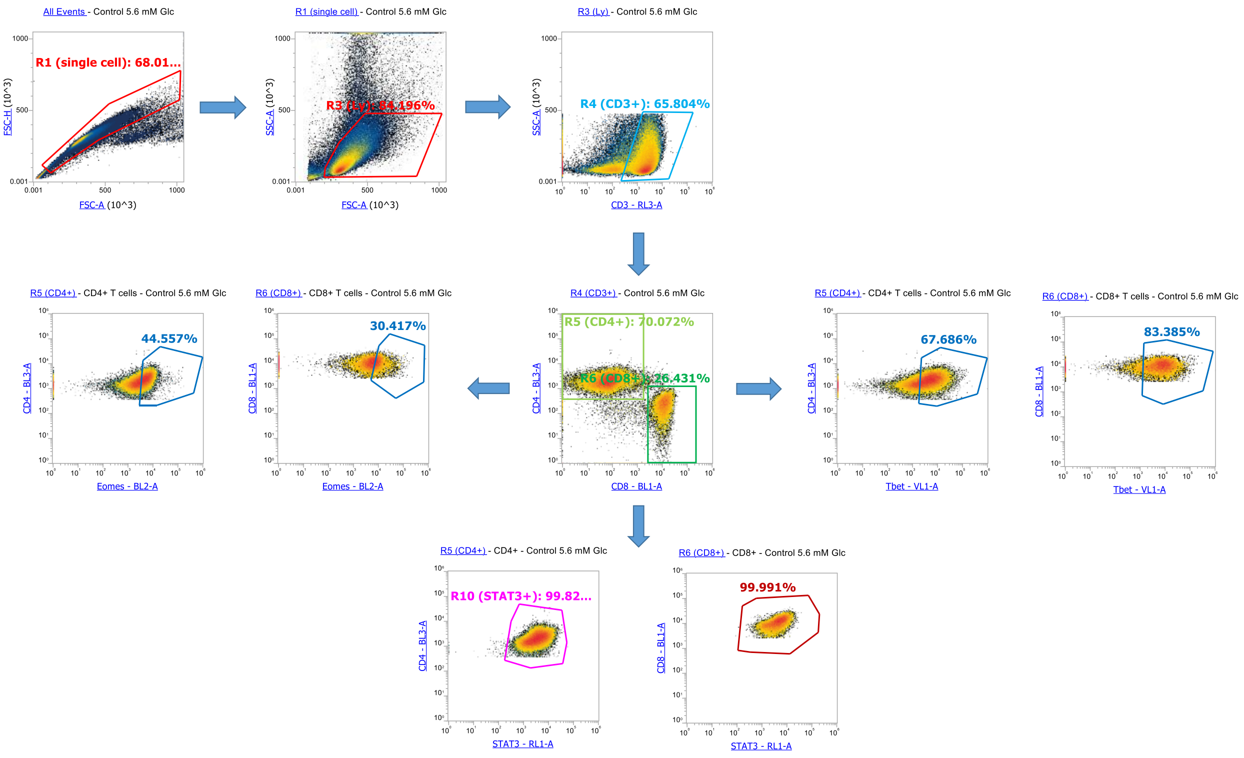

The Eomes+, T-bet+ and STAT3+ gates were set according to FMO controls.

1.2.6.2 HIF-1α and P-S6RP expression

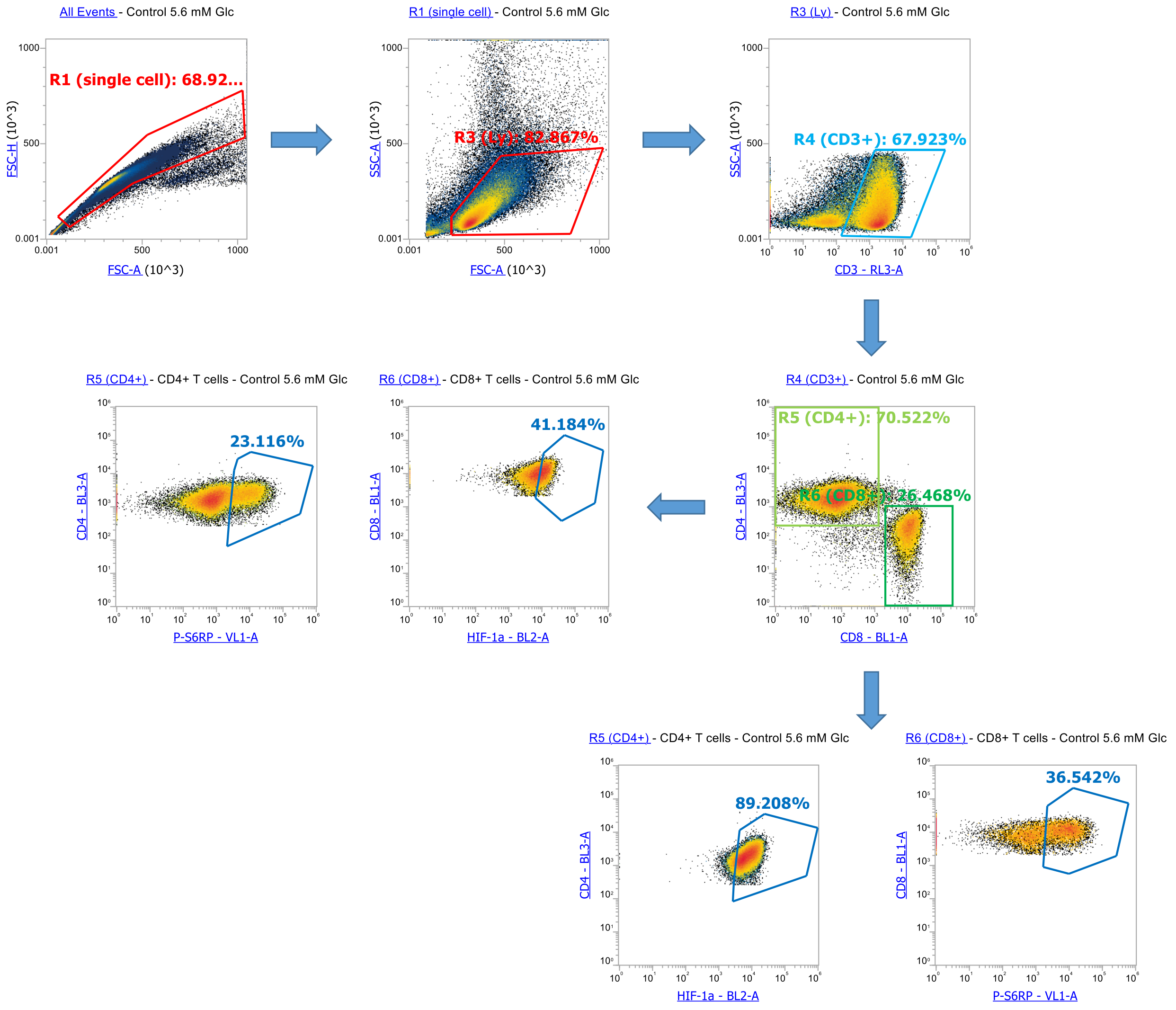

The P-S6RP+ and HIF-1α+ gates were set according to FMO controls.

2 References

1. Repas J, Zupin M, Vodlan M, Veranič P, Gole B, Potočnik U, et al. Dual Effect of Combined Metformin and 2-Deoxy-D-Glucose Treatment on Mitochondrial Biogenesis and PD-L1 Expression in Triple-Negative Breast Cancer Cells. Cancers. 2022 Jan;14(5):1343.
